# How Much Food Can We Grow in Urban Areas? Food Production and Crop Yields of Urban Agriculture: A Meta‐Analysis

**DOI:** 10.1029/2022EF002748

**Published:** 2022-08-26

**Authors:** Florian Thomas Payen, Daniel L. Evans, Natalia Falagán, Charlotte A. Hardman, Sofia Kourmpetli, Lingxuan Liu, Rachel Marshall, Bethan R. Mead, Jessica A. C. Davies

**Affiliations:** ^1^ Lancaster Environment Centre Lancaster University Lancaster UK; ^2^ Centre for Soil, Agrifood and Biosciences Cranfield University Cranfield UK; ^3^ Department of Psychology University of Liverpool Liverpool UK

**Keywords:** urban food growing, urban spaces, growing systems, agricultural productivity, food security, urban resilience

## Abstract

Urban agriculture can contribute to food security, food system resilience and sustainability at the city level. While studies have examined urban agricultural productivity, we lack systemic knowledge of how agricultural productivity of urban systems compares to conventional agriculture and how productivity varies for different urban spaces (e.g., allotments vs. rooftops vs. indoor farming) and growing systems (e.g., hydroponics vs. soil‐based agriculture). Here, we present a global meta‐analysis that seeks to quantify crop yields of urban agriculture for a broad range of crops and explore differences in yields for distinct urban spaces and growing systems. We found 200 studies reporting urban crop yields, from which 2,062 observations were extracted. Lettuces and chicories were the most studied urban grown crops. We observed high agronomic suitability of urban areas, with urban agricultural yields on par with or greater than global average conventional agricultural yields. “Cucumbers and gherkins” was the category of crops for which differences in yields between urban and conventional agriculture were the greatest (17 kg m^−2^ cycle^−1^ vs. 3.8 kg m^−2^ cycle^−1^). Some urban spaces and growing systems also had a significant effect on specific crop yields (e.g., tomato yields in hydroponic systems were significantly greater than tomato yields in soil‐based systems). This analysis provides a more robust, globally relevant evidence base on the productivity of urban agriculture that can be used in future research and practice relating to urban agriculture, especially in scaling‐up studies aiming to estimate the self‐sufficiency of cities and towns and their potential to meet local food demand.

## Key Points

- A meta‐analysis was conducted to quantify crop yields of urban agriculture and investigate how they compare with conventional agriculture
- Urban areas have high agronomic suitability, with urban agricultural yields on par with or greater than conventional agricultural yields
- Yields differ significantly between certain urban spaces and growing systems where crops are grown, which shows varying crop suitability

## 1 Introduction

Population growth, dietary changes and growing bioeconomies are placing unprecedented pressure on global food and land systems (Davis et al., 2014). The rising food demands associated with growing populations and transitioning diets are likely to become harder to meet as agricultural production rates are expected to reach a plateau during the 21st century (Ray et al., 2012). The increasing use of biofuels and other bio‐based materials is competing for agricultural land use and resources (Muscat et al., 2020; Rathmann et al., 2010), while climate change impacts are predicted to increase the variability of food production and uncertainty in crop yields (Mbow et al., 2019). The intensification of international trade has also led to the globalization of food commodities and to an increased disconnection between human populations and the land and water resources that support food production (D’Odorico et al., 2014). The impact of future disruptions (e.g., climate change or global pandemics) on food supply chains will especially affect countries heavily reliant on imports such as the United Kingdom (Bren d’Amour et al., 2016; Garnett et al., 2020; Yoshida & Yagi, 2021).

Our current food system is also facing pressing challenges to reduce its ecological footprint. The agricultural sector is currently a major contributor to climate change and environmental degradation. It is estimated that between 21% and 37% of total anthropogenic greenhouse gas emissions are linked to the global food system (IPCC, 2019; Lynch et al., 2021; Mbow et al., 2019; Rosenzweig et al., 2020). Modern agricultural practices account for more than 70% of water withdrawals at the global level and contribute to the acidification and eutrophication of aquatic and terrestrial ecosystems via the intensified use of agrochemicals (Clark & Tilman, 2017). Land expansion for food production is also associated with widespread deforestation, soil degradation, biodiversity loss and habitat fragmentation (Olsson et al., 2019; Zabel et al., 2019).

Urban food growing has been identified as one potential solution to the challenges our global food system is facing (Clinton et al., 2018; Walsh et al., 2022). In recent years, various definitions have been proposed by the academic literature to define what urban agriculture is and encompasses (e.g., Ackerman et al., 2014; Colasanti et al., 2012; Ghisellini & Casazza, 2016; Miccoli et al., 2016; Mougeot, 2001; Pearson et al., 2010; Smit et al., 2001). But at the core of all these definitions is the understanding that urban agriculture involves food production in urban areas (Opitz et al., 2016). In this study, we refer to urban agriculture as the production, processing and marketing of food on all types of publicly and privately held land and water bodies dispersed throughout urban and peri‐urban areas, mostly destined to consumers residing in these areas (Pearson et al., 2010; Smit et al., 2001).

Urban agriculture currently plays an important role in the global food system and studies have highlighted the potential for urban agriculture to contribute further to food security, food system resilience and sustainability (Opitz et al., 2016; O’Sullivan et al., 2019; Walsh et al., 2022). It is estimated that between 5% and 10% of the global production of legumes, vegetables and tubers is currently delivered by urban agriculture (Clinton et al., 2018), while between 15% and 20% of global food is produced in urban and peri‐urban environments (Abdulkadir et al., 2012). Utilizing the food production potential of global peri‐urban areas only could locally nourish approximately 30% of the global urban population, though with variations between different regions of the world (Kriewald et al., 2019). Several studies (e.g., Astee & Kishnani, 2010; Despommier, 2011; Mendes, 2008) have also shown that urban agriculture improves the provision of fresh food in cities and enhances urban populations' access to locally grown food. This could prove particularly useful in “food deserts,” that is to say neighborhoods in high‐income countries with limited fresh food retail (Raja et al., 2008; Specht et al., 2014). Moreover, urban food production is expected to be more resilient than conventional agriculture because of its short supply chain for urban dwellers and diversified farming activities (Yoshida & Yagi, 2021). Urban agriculture can also contribute to mitigating the negative effects of future food system disruptions. In terms of environmental sustainability, urban food growing delivers a wide range of ecosystem services, including regulating (e.g., carbon sequestration, reducing air pollution), provisioning (e.g., food production, medicinal resources), supporting (e.g., biodiversity, soil formation) and cultural (e.g., well‐being, better diet quality) services (Clinton et al., 2018; Evans et al., 2022; Mead et al., 2021; Russo et al., 2017; Small et al., 2019). Urban agriculture may also contribute to global efforts in mitigating climate change, though there is still uncertainty as to whether the carbon footprint of urban agriculture is indeed lower than that of conventional agriculture for provisioning urban areas. Although urban food production is being promoted as a means for cities and towns to decrease food miles by shortening the supply chain (Benis et al., 2020; Specht et al., 2014), which would reduce food transport emissions (Pradhan et al., 2020), assessments of greenhouse gas emissions associated with food production have shown very mixed results, proving that local food production does not necessarily lead to lower emissions (Coelho et al., 2018). While some studies (e.g., Dyer et al., 2011; Payen et al., 2015) showed that increasing self‐sufficiency at the city level through local production was more carbon‐intensive than the current practice of importing certain crops, others (e.g., Benis et al., 2017, 2018; Dorr et al., 2021) proved that urban growing systems could be associated with a lower global warming potential than current supply chains, depending on the crops and countries considered.

Despite the growing literature on urban food production, our understanding of urban agriculture is in its relative infancy when compared to conventional rural contexts, and there is a need for synthesizing our current knowledge (Artmann et al., 2021). Several studies (e.g., Armanda et al., 2019; O’Sullivan et al., 2019) have reported the absence of literature on empirical and global crop yield and production data for urban agriculture, resulting in continued doubts about the yield and food supply capacity of urban agriculture (Yan et al., 2022). To our knowledge, there is no global assessment evaluating crop productivity and yields of urban agriculture. The meta‐analysis by Dorr et al. (2021) reported urban agricultural yields for a variety of crops grown in different urban spaces, but their study aimed to conduct a meta‐analysis of life cycle assessments of urban agriculture, of which crop yields were a component, and not a meta‐analysis of crop yields per se. The overall number of observations for crop yields was, consequently, somewhat low in their study (*n* = 125). As a result of this lack of systematic and integrative assessment of crop yields of urban agriculture, scaling‐up studies aiming at quantifying the productive potential of cities and towns tend to use yield values from conventional agriculture, field experiments conducted in specific urban spaces at the local level or particular agri‐businesses in their analysis (e.g., Gondhalekar & Ramsauer, 2017; Grewal & Grewal, 2012; Haberman et al., 2014; Hsieh et al., 2017; Saha & Eckelman, 2017; Walsh et al., 2022), which may create inaccurate estimates of the extent to which urban agriculture could meet the food demand of urban populations and contribute to making urban areas self‐sufficient (Weidner et al., 2019).

In addition, the focus in the literature on urban agriculture has been put on green spaces (e.g., private gardens, allotments, parks) and relatively little attention has been paid to gray spaces (i.e., spaces that have been artificialized and replaced by impermeable materials known as “gray infrastructure”) because these spaces are at the edge of our food growing frontier (Evans et al., 2022; Walsh et al., 2022). Though interest is rising, the examination of the productive potential of these spaces, their agronomic suitability, and how their food production could fit within the wider food system remains relatively under‐studied. It is still unclear what types of crops can be grown using gray spaces and in which quantity. Furthering our understanding of the potential of urban spaces for food production, and particularly estimating crop yields that could be reached in urban environments, is crucial to better design urban agricultural systems and plan how urban food growing could contribute to reducing food insecurity in cities and towns.

In this meta‐analysis, we assessed the agricultural productivity of urban systems by estimating global values of crop yields obtained in urban environments. We aimed to address the following research questions: (a) What types of crops can be successfully grown in urban environments and in what quantity? (b) How does urban agricultural productivity compare with the productivity of conventional agriculture? and (c) What is the agronomic suitability of urban gray spaces and to what extent could these spaces contribute to urban food production? In answering these questions, we expected to provide the largest globally relevant evidence base for the productivity of urban agriculture, which could support further research investigating the potential of cities and towns to help to create a more sustainable, equitable and resilient food system. This paper also intended to identify the distinct characteristics of urban spaces and growing systems that yield the highest crop productivity, which can support future urban agriculture research and practice.

## 2 Materials and Methods

### 2.1 Data Collection

**Figure 1:**
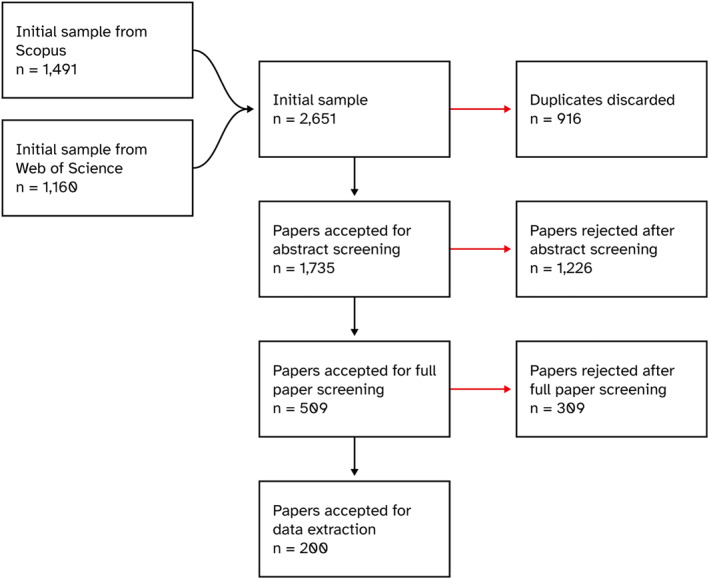
PRISMA flowchart indicating the different stages and outputs of the data screening in this meta‐analysis.

A literature search was conducted in May 2021 to identify peer‐reviewed publications reporting crop yields in urban and peri‐urban environments at the global level. To ensure an optimal reporting of the relevant publications for this study, we followed the Preferred Reporting Items for Systematic reviews and Meta‐Analyses (PRISMA) protocol as described in Moher et al. (2009, 2015). Figure 1 illustrates the different steps undertaken to compile the final sample of studies selected for analysis.

The search was performed on the electronic databases of Scopus and Web of Science using the following search string:

(“urban agricultur*” OR “urban horticultur*” OR “urban food grow*” OR “urban farm*” OR “city agricultur*” OR “city horticultur*” OR “city food grow*” OR “city farm*” OR “building‐integrated agricultur*” OR “zero acreage farm*” OR “skyfarm*” OR “sky garden*” OR “vertical farm*” OR “controlled environment agricultur*” OR “rooftop farm*” OR “rooftop garden*” OR “rooftop greenhouse*” OR “shipping container farm*” OR “indoor farm*” OR “indoor agricultur*” OR “edible wall*” OR “edible façade*” OR ((“urban” OR “city”) AND (“greenhouse*” OR “hydroponic*” OR “aeroponic*” OR “aquaponic*”))) AND (“yield*” OR “food producti*”)

These search terms had to feature in the title, abstract or keywords of the publications. They were refined to ensure that results included the key papers reporting crop yields of urban agriculture. The first section of the search string narrows down results to agriculture and food growing taking place in urban and peri‐urban areas and specifies gray urban spaces and food growing systems for which the terms “urban agriculture” or “urban food growing” may not necessarily be mentioned in the publication; the second section aims to limit results to studies focusing on the productive aspect of urban agriculture. Food growing spaces that are de facto located in urban or peri‐urban environments (e.g., indoor farming) were used without the terms “urban” or “city” in the search string, while those that could be located in either rural or urban areas (e.g., greenhouses) were used in conjunction with “urban” or “city." Only peer‐reviewed studies (conference proceedings or scientific papers) published in or after 2000 were searched. This initial search yielded 2,651 results; of these, 916 results were duplicates and were, therefore, discarded, resulting in 1,735 publications accepted for abstract screening.

Three criteria were used to screen the abstracts: (a) the study needed to deal with one or several crops grown for food consumption (livestock products and biofuel crops were excluded from the analysis); (b) the study area had to be urban or peri‐urban, considering peri‐urban those transitional zones in the margin of cities that have been occupied to develop environmental services, which result from the dynamic interaction between rural and urban systems (Dadashpoor & Somayeh, 2019; Rauws & De Roo, 2011); and (c) the abstract needed to refer to an urban food growing space (e.g., “allotment” or “rooftop garden”). After the abstract screening, 1,226 papers were further rejected, resulting in 509 publications being accepted for full‐text screening. Three additional criteria were used to screen the papers: (d) the study had to report at least one yield value; (e) yield values needed to be reported for a specific crop or group of crops (yields per growing space, such as the yield per plot, were excluded); and (f) yields had to be measured empirically (yields from modeling studies or obtained by extrapolation were excluded). 309 publications were further rejected after the full‐text screening phase. The final sample for data extraction was made of 200 publications.

### 2.2 Data Extraction

For each paper, information on urban agricultural yields was collected and organized in an Excel sheet. Each row corresponded to a different observation (i.e., a yield value). Yields corresponded to the fresh weight of a harvested crop for a given area in a given year (except for pulses, where it corresponded to the dry weight of the crop harvested instead).

In a certain number of studies, the yield was not directly available and the yield per plant was reported instead. In such cases, we estimated the crop yield using the crop cut method (Sapkota et al., 2016), following Equation 1. We ensured that the size of the sampling plots was at least 1 m^2^ to minimize bias.

(1) $$\text{Yield}\;\left({\text{weight}\;\text{area}}^{-1}\;{\text{yr}}^{-1}\right)=\text{yield}\;\text{per}\;\text{plant}\;\left({\text{weight}\;\text{plant}}^{-1}\;{\text{yr}}^{-1}\right)\times \text{number}\;\text{of}\;\text{plants}\;\text{grown}\;\text{in}\;a\;\text{given}\;\text{area}\;\left({\text{area}}^{-1}\right)$$

Most reported yield values corresponded to one growing cycle per year; however, some studies took into account several cycles per year (up to 12 in some cases). To ensure that all yield values could be compared without biases, we divided the yield per year by the number of growing cycles and used the yield per cycle (weight area^−1^ cycle^−1^) for analysis instead. For harmonization purposes, all yield values were expressed in kg m^−2^ cycle^−1^.

For each yield value, we collected data on the type of crop grown (e.g., lettuce, cabbage, strawberry, etc.), the growing methods used (e.g., soil‐based, fertilizer use, pot experiment, etc.), the production system used (e.g., greenhouse, urban farm, allotment, etc.), the urban space where the crop was grown (e.g., rooftop, façade, indoor, etc.), the town or city and country where the crop was grown, details on yield calculations (i.e., the number of replications and years used), and the number of growing cycles per year considered in the yield measurements. This information was available for each yield value. All collected information is stored in a database available in Data Set S1.

### 2.3 Definition of Categories

To facilitate analysis, the information extracted for each yield value was used to classify observations based on the type of crop grown, the urban space used for food production, and the characteristics of the growing system.

#### 2.3.1 Crop Grown

The categories developed by the Food and Agriculture Organization of the United Nations (FAO, 2022) and available on their online platform FAOSTAT to report global agricultural yields were used to group observations by crop grown in this study. This made our findings on urban agricultural yields easily comparable to yields from conventional agriculture published by the FAO (2022). Each observation was classified by crop grown twice, once according to the FAO aggregated items list and once according to the FAO disaggregated items list. We found yield values for eight of the FAO aggregated items list: “cereals,” “fiber crops primary,” “fruit primary,” “oilcrops,” “pulses,” “roots and tubers,” “sugar crops primary,” and “vegetables primary.” Disaggregated items from the FAO list for which yield values were found are presented in Table 1.

**Table 1:** List of the Categories Used to Group Crops Based on the FAOSTAT Database (FAO, 2022)

Aggregated crop categories	Disaggregated crop categories	Crops included in the categories (for which observations were found)
Cereals (*n* = 321)	Barley (*n* = 5)	Barley
	Cereals nes (*n* = 16)	Amaranth
	Maize (*n* = 93)	Maize
	Millet (*n* = 2)	Millet
	Paddy rice (*n* = 124)	Paddy rice
	Quinoa (*n* = 8)	Quinoa
	Sorghum (*n* = 11)	Sorghum
	Wheat (*n* = 62)	Wheat
Fiber crops primary (*n* = 13)	Bastfibers, other (*n* = 4)	Roselle
	Jute (*n* = 9)	Jute mallow
Fruit primary (*n* = 98)	Apples (*n* = 2)	Apples
	Avocados (*n* = 1)	Avocados
	Bananas (*n* = 1)	Bananas
	Berries nes (*n* = 4)	Blackberries, ginseng berries
	Blueberries (*n* = 1)	Blueberries
	Cherries (*n* = 1)	Cherries
	Currants (*n* = 2)	Currants
	Fruit, tropical fresh nes (*n* = 1)	Dragon fruit
	Gooseberries (*n* = 1)	Gooseberries
	Mangoes, mangosteens, guavas (*n* = 1)	Guavas
	Melons, other (including cantaloupes) (*n* = 2)	Melons
	Papayas (*n* = 1)	Papayas
	Peaches and nectarines (*n* = 1)	Peaches
	Pears (*n* = 2)	Pears
	Persimmons (*n* = 1)	Persimmons
	Plums and sloes (*n* = 3)	Plums
	Raspberries (*n* = 1)	Raspberries
	Strawberries (*n* = 70)	Strawberries
	Watermelons (*n* = 2)	Watermelons
Oilcrops (*n* = 28)	Rapeseed (*n* = 16)	Oil‐seed rape
	Soybeans (*n* = 12)	Soybeans
Pulses (*n* = 1)	Chickpeas (*n* = 1)	Chickpeas
Roots and tubers (*n* = 24)	Potatoes (*n* = 14)	Potatoes
	Roots and tubers nes (*n* = 4)	Jerusalem artichokes
	Sweet potatoes (*n* = 6)	Sweet potatoes
Sugar crops primary (*n* = 8)	Sugar beet (*n* = 8)	Sugar beets
Vegetables primary (*n* = 1,569)	Anise, badian, fennel, coriander (*n* = 24)	Caraway, coriander, fennel (seeds)
	Artichokes (*n* = 1)	Artichokes
	Asparagus (*n* = 1)	Asparaguses
	Aubergines (*n* = 37)	Aubergines
	Beans (*n* = 85)	Common beans, string beans
	Cabbages and other brassicas (*n* = 238)	Brussel sprouts, cabbages, collards, kales, kohlrabies, leaf mustards, pak choi
	Carrots and turnips (*n* = 41)	Carrots, turnips
	Cauliflowers and broccoli (*n* = 134)	Cauliflowers, broccolis
	Chillies and peppers (*n* = 69)	Chilli peppers, bell peppers
	Cucumbers and gherkins (*n* = 58)	Cucumbers
	Garlic (*n* = 13)	Garlics
	Leeks and other alliaceous vegetables (*n* = 3)	Leeks, chives
	Lettuce and chicory (*n* = 344)	Lettuces, chicories, endives, mesclun
	Okra (*n* = 15)	Okras
	Onions and shallots (*n* = 20)	Onions, shallots, spring onions
	Peas (*n* = 8)	Green peas, mangetout peas
	Peppermint (*n* = 1)	Peppermint
	Pumpkins, squash and gourds (*n* = 37)	Courgettes, pumpkins, squashes
	Spices nes (*n* = 1)	Dill
	Spinach (*n* = 29)	Spinaches
	Tomatoes (*n* = 208)	Cherry tomatoes, tomatoes
	Vegetables, fresh nes (*n* = 202)	Basil, beetroots, celeriac, celeries, Swiss chards, fennel (bulb), marjoram, parsnips, radishes, rhubarbs, water spinaches, watercresses

*Note.* Categories with “nes” correspond to all the crops not included in other categories of the same crop type (as they do not have much relevance at the global level). “Fresh” is used by the FAOSTAT to specify that the category refers to non‐processed crops (although all the categories presented here refer to non‐processed crops). The number of observations for each category appears in brackets.

#### 2.3.2 Urban Space

Observations were also classified depending on the urban space used to produce the crop (Table 2). We developed two broad categories: “gray spaces” and “green spaces.”

1. The category “gray spaces” corresponds to urban food production located on artificialized land or systems deploying zero‐acreage farming, that is to say food production characterized by the non‐use of land or acreage for farming activities (Thomaier et al., 2015).
2. The category “green spaces” refers to urban food growing taking place in urban vegetated spaces, that is to say vegetated spaces traditionally located within built‐up areas (such as allotments, parks, community and private gardens, yards, and urban farms), and in “natural” environments, that is to say areas of vegetation or bodies of water in an urban landscape, including forests, coastal areas, riparian spaces and wilderness areas (Taylor & Hochuli, 2017).

**Table 2:** List of the Categories Used to Classify Observations per Urban Space

Urban space		Definition	Includes
Gray spaces	Façades	Urban food production located on buildings' façades	Green walls, suspended balconies
	Ground	Urban food production taking place on ground‐based urban land, that is to say land that is not located on or within a building and that is not classified as a green space	Brownfields, vacant lots, parking areas, roadside and pathways, school and university grounds, religious spaces
	Indoor	Urban food production located within existing buildings	Plant factories, growth chambers, offices, private flats and houses
	Rooftops	Urban food production taking place on buildings' rooftops	Rooftop gardens, rooftop farms, rooftop‐integrated greenhouses
Green spaces		Urban food production taking place in urban vegetated spaces traditionally located within built‐up areas and in “natural” environments, that is to say areas of vegetation or bodies of water located in an urban landscape	Allotments, parks, community and private gardens, yards, urban farms, forests, coastal areas, riparian spaces, wilderness areas

The category “gray spaces” was further divided into four sub‐categories: “façades,” “ground,” “indoor,” and “rooftops.” These sub‐categories were created so that urban spaces with important differences in terms of location, infrastructures, agricultural practices, inputs, etc. could be distinguished since the specific characteristics of these spaces may influence the type of crops that they can accommodate along with their potential for food production.

1. The sub‐category “façades” corresponds to food production taking place on buildings’ façades, such as green walls or suspended balconies.
2. “Ground” refers to ground‐based food growing (i.e., not on or within a building) taking place on urban land that is not classified as a green space; types of spaces categorized as “ground” include brownfields, vacant lots, parking areas, roadside and pathways, school and university grounds, and religious spaces. Separating “ground” spaces from “green spaces” allows us to investigate the agronomic suitability and agricultural productivity of ground‐based “gray spaces” specifically, for which the existing knowledge is less developed, and to compare them with those of “green spaces.”
3. “Indoor” represents urban agriculture located within existing buildings, such as plant factories, growth chambers, offices or even private flats and houses.
4. “Rooftops” relates to food production taking place on buildings’ rooftops, such as rooftop gardens, rooftop farms or rooftop‐integrated greenhouses.

These categories and sub‐categories of urban spaces considered solely where the food production took place, that is to say in which urban space, and were not at all based on how the food was produced, which falls under the type of growing system used. For instance, the category “rooftop” only means that the crop was grown on top of a building but does not give any information as to how: it could have been in an open‐air rooftop garden or in a rooftop greenhouse, using hydroponics or a soil‐based system, etc. Similarly, a raised bed could be located in an allotment and be classified as “green space” or in a brownfield and be classified as “ground” space or in a rooftop garden and be classified as “rooftop,” etc. The only category of urban space that intrinsically gives information on how the crop was produced is “indoor” due to the fact that crops produced inside a building are most likely cultivated in a controlled‐environment system.

#### 2.3.3 Growing System

We were interested in the effect of three aspects of the growing system on yields: whether farming was conducted vertically or horizontally, the type of medium used to grow the crop and the level of conditioning of the environment the crop was grown in. We created categories for each of these aspects, which are described in more detail below.

Two categories were created to reflect whether farming was conducted vertically or horizontally: “vertical farming” and “horizontal farming.” Though the notion of vertical farms is often associated with systems located indoors, with fully artificial lighting and using hydroponic methods (e.g., Avgoustaki & Xydis, 2020a), the category “vertical farming” used in this study was much broader and included any type of systems where several layers of crops were grown in a given area (e.g., green walls, multi‐layered greenhouses, indoor plant factories, etc.). “Horizontal farming” refers to conventional, horizontally oriented food production systems, in which a single layer of crops is cultivated in a given area.

For the growing medium, two categories were created: “hydroponic systems” and “soil‐based systems.” The “hydroponic systems” category gathers both hydroponic and aquaponic systems, that is to say systems where the growing substrate, whether organic (such as coconut coir) or inorganic (such as perlite), does not provide nutrients to the crop. Nutrients are delivered, instead, using a nutrient solution that is regularly or even continuously applied to the crop. The number of observations taking place in aquaponic systems (*n* = 18) was too small to be used as a distinct category and successfully compared to other systems. “Soil‐based systems” refers to systems where crops are provided with the nutrients required for their growth directly via the substrate that they grow in (with the optional support of fertilizers to complement the nutrient availability of the substrate). No aeroponic system was found in the literature search.

Regarding the level of conditioning of the growing system, three categories were created: “controlled‐environment agriculture with sunlight,” “controlled‐environment agriculture with artificial light,” and “open‐air agriculture.” Controlled‐environment agriculture is a technology‐based approach to food production that consists of protecting crops from outdoor elements to maintain optimal growing conditions throughout their developmental cycle (Lefers et al., 2020; Shamshiri et al., 2018). The category “controlled‐environment agriculture with sunlight” gathers controlled‐environment systems (such as polytunnels and greenhouses) where the lighting is provided naturally via sunlight, most often thanks to see‐through walls and roofs or see‐through polyethene covering. Some of these systems also have supplementary lighting to extend daylight hours. “Controlled‐environment agriculture with artificial light” corresponds to controlled‐environment systems (such as growth chambers or plant factories) where no natural lighting reaches the crop and artificial lighting (via light‐emitting diodes, for instance) is solely used instead. All the observations in this category were located in indoor spaces. Though controlled‐environment agriculture is usually associated with the use of soilless cultivation methods such as hydroponics, aeroponics or aquaponics (Ragaveena et al., 2021), soil‐based methods were also found to be used in such environments, particularly in greenhouses and indoor farms. “Open‐air agriculture” refers to agricultural systems where crops are cultivated in the open and are not physically protected from outdoor elements (e.g., fields, gardens, rooftop terraces, etc.).

The categories created for each aspect of the growing system investigated in this paper were solely based on how the food was produced and not at all on where, contrary to the categories created for urban spaces. For example, a controlled‐environment system could have been set up on the ground, on a rooftop or inside a building.

### 2.4 Meta‐Analysis

This meta‐analysis aimed to estimate the mean value of crop yields reached in urban food growing systems for different crops or groups of crops. We used the arithmetic mean of yield values (AM_y_) as an effect size in the meta‐analysis since the operationalization of the variable (i.e., yield value) was the same for all crops or groups of crops. Using arithmetic means in meta‐analyses is an effective way to gauge a quantity's magnitude and allows for easy comparisons between different groups (Johnson & Eagly, 2014). The effect size AM_y_ was calculated according to the methods of Lipsey and Wilson (2001) following Equation 2, where *x*_*i*_ corresponds to the yield value (in kg m^−2^ cycle^−1^) reported for observation *i* (*i* = 1 to *n*) and *n* is the sample size.

(2) $${\text{AM}}_{y}=\bar{X}=\;\frac{\sum x_{i}}{n}$$

Because yields vary intrinsically based on the type of crops, an effect size was calculated for each category of crop grown (for both disaggregated and aggregated categories) rather than an overall effect size from all the observations in the data set. The arithmetic mean was not weighted for either of the category groups, which means that each observation had the same weight in the calculation process and, therefore, the same influence on the effect size. Effect sizes could not be weighted by the inverse of the variance due to most studies in the data set not reporting variability (Smith et al., 2019). We also decided not to weight effect sizes using the sample size as studies conducted on experimental plots often used small plots with extensive replication, whereas studies taking place on actual farms (where crops were cultivated by urban farmers or gardeners instead of researchers) tended to be conducted at larger spatial scales but with lower replication. Using a weighted effect size would have given experimental plots markedly greater weight than on‐farm studies, which are often, however, more representative of real‐world urban farming conditions (Crowder & Reganold, 2015). Bias‐corrected 95% confidence intervals were generated for each effect size computed using a bootstrapping procedure with 10,000 iterations (Adams et al., 1997).

The impact of different urban spaces and the level of conditioning of the growing system on crop yields was assessed by one‐way analysis of variance (ANOVA), followed by a post hoc Tukey's honest significant difference (HSD) test when the results of the ANOVA suggested significance at the 95% confidence level (Allory et al., 2021; Hu et al., 2017). Variations in crop yields between vertical and horizontal farming and between hydroponic and soil‐based systems were tested using independent two‐sample *t*‐tests (assuming unequal variances) at the 95% confidence level (Mathew et al., 2020). Boxplots were used to visualize data distribution and variability. All the analyses were conducted in SPSS 27 (IBM Corp, 2020).

### 2.5 Yields of Conventional Agriculture

To investigate how urban agricultural yields compared to conventional agricultural yields, we used the global average yields of conventional agriculture from the FAOSTAT database (FAO, 2022). These global average yields of conventional agriculture are calculated by the FAO for a given year and a specific crop category (aggregated or disaggregated) as the sum of the crop production quantity of each country divided by the sum of the area harvested for the crop category in each country, using a mix of official, semi‐official, estimated or calculated data. They provide the most accurate estimate of the average yields of conventional agriculture at the global level and include all the different ways conventional agriculture is conducted globally (e.g., open‐air fields vs. greenhouses). To control for yearly variations in yields due to weather conditions and other factors (e.g., ban of neonicotinoids for oilseeds), we computed the average of these yearly global average yields for the period 2015–2020 for each aggregated and disaggregated category of crops considered in this meta‐analysis and used these values for our comparisons between urban and conventional agricultural yields. 95% confidence intervals were generated for each crop category to account for the high variability of yields between countries.

## 3 Results

### 3.1 General Findings

Screening identified 200 studies eligible for inclusion, from which we extracted 2,062 observations of urban agricultural yield values. An overview of the studies included in this meta‐analysis can be found in Supporting Information S1. Most of the studies were published between 2014 and the first half of 2021 (*n* = 152). The number of studies reporting urban agricultural yields increased over the past couple of years, with 85 studies out of 200 having been published between 2019 and the first half of 2021 only.

We found observations for 8 aggregated categories of crops (Table 1). Vegetables were by far the most represented types of crops in our data set (*n* = 1,569), followed by cereals (*n* = 321), fruit (*n* = 98), oil crops (*n* = 28), roots and tubers (*n* = 24), fiber crops (*n* = 13), sugar crops (*n* = 8) and, finally, pulses (*n* = 1). We also found observations for 58 disaggregated categories of crops (Table 1). “Lettuce and chicory” was the disaggregated category of crops with the highest number of observations in the sample (*n* = 344), followed by “cabbages and other brassicas” (*n* = 238), “tomatoes” (*n* = 208), “vegetables, fresh nes” (*n* = 202), “cauliflowers and broccoli” (*n* = 134), “paddy rice” (*n* = 124), “maize” (*n* = 93), “beans” (*n* = 85), “strawberries” (*n* = 70), “chillies and peppers” (*n* = 69), “wheat” (*n* = 62), and “cucumbers and gherkins” (*n* = 58). The rest of the disaggregated categories of crops had fewer than 50 observations.

**Figure 2:**
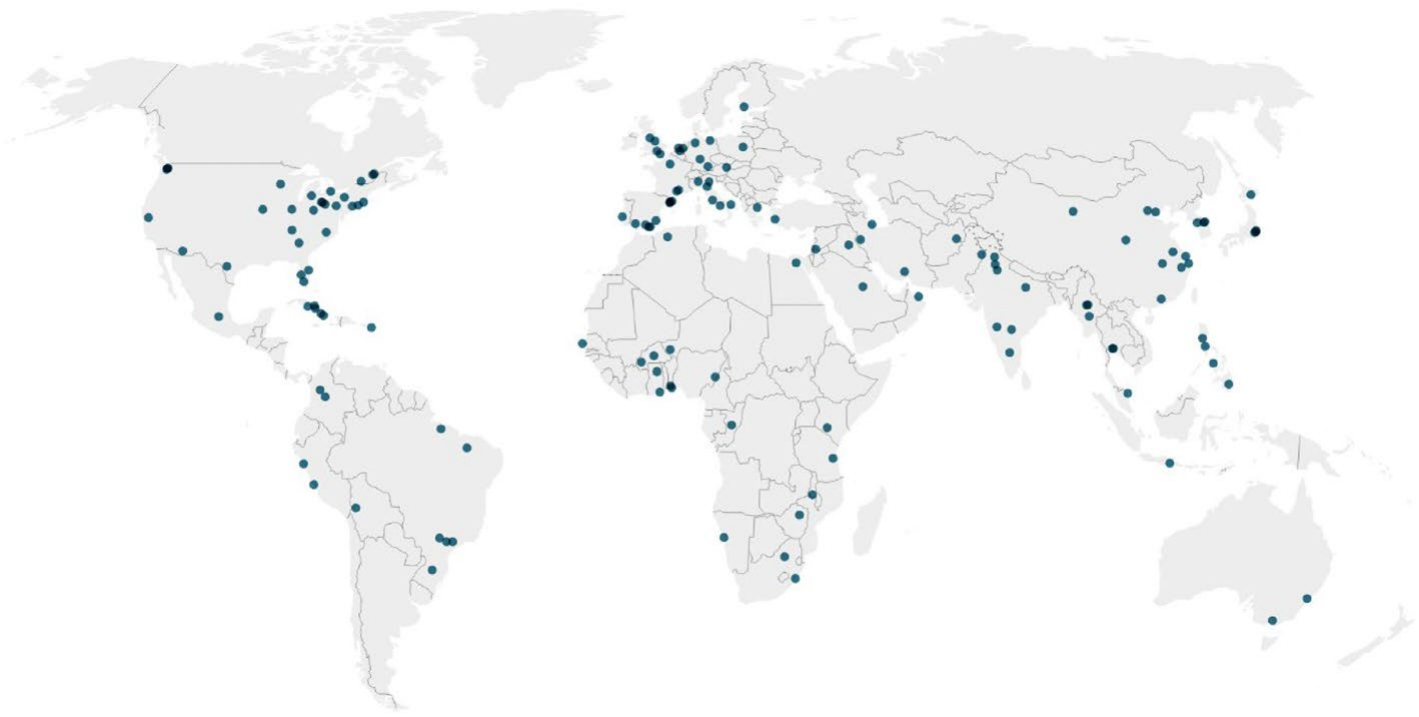
Global distribution of the cities and towns where urban agriculture was conducted in our data set. Each dot represents a city or town where field experiments took place. The color of the dots does not reflect the number of field experiments coming from each city or town; darker colors are a consequence of dots overlapping when several cities or towns are too close to each other.

Overall, our data set gathered observations from a multitude of cities and towns located in various countries and regions of the world (Figure 2). The majority of the observations in the data set were located in Europe (*n* = 516), while the rest of the observations took place in North America (*n* = 398), East Asia (*n* = 361), Sub‐Saharan Africa (*n* = 223), South America (*n* = 161), South Asia (*n* = 151), Southeast Asia (*n* = 131), the Middle East (*n* = 68), Central America (*n* = 25), Oceania (*n* = 24) and, finally, North Africa (*n* = 4). Fifty‐three different countries were represented in the data set, with the largest number of observations conducted in the United States of America (*n* = 349), followed by Japan (*n* = 189), China (*n* = 166), Spain (*n* = 126), India (*n* = 120), Brazil (*n* = 110) and Italy (*n* = 103). The number of observations for the rest of the countries was lower than 100. We found observations for 147 distinct cities and towns in total. Tsukuba, Japan was the most represented city in the sample, with 120 yield values gathered, followed by Paris, France (*n* = 82) and Bologna, Italy (*n* = 67).

Observations were found for several different types of urban agricultural spaces. Urban green spaces represented the majority of observations in the data set (*n* = 1,214), while approximately 40% of the observations were for urban gray spaces (*n* = 848). Among gray spaces, the “ground” sub‐category was the most represented, with 379 observations, followed by rooftops (*n* = 250) and indoor spaces (*n* = 208). The number of observations for the sub‐category "façades" was, by comparison, much lower (*n* = 11). The vast majority of observations corresponded to horizontal farming (*n* = 1,853), whereas vertical farming was considerably less prominent in the data set (*n* = 209). Soil‐based systems represented about 73% of all observations in the data set (*n* = 1,512), while 27% of observations took place in hydroponic systems (*n* = 550). Finally, most observations happened in open‐air environments (*n* = 1,194) and in controlled‐environment spaces with sunlight (*n* = 660). Controlled‐environment agriculture with artificial light applied to only 10% of the observations (*n* = 208).

### 3.2 Productivity of Urban Agriculture Compared to Conventional Agriculture

**Figure 3:**
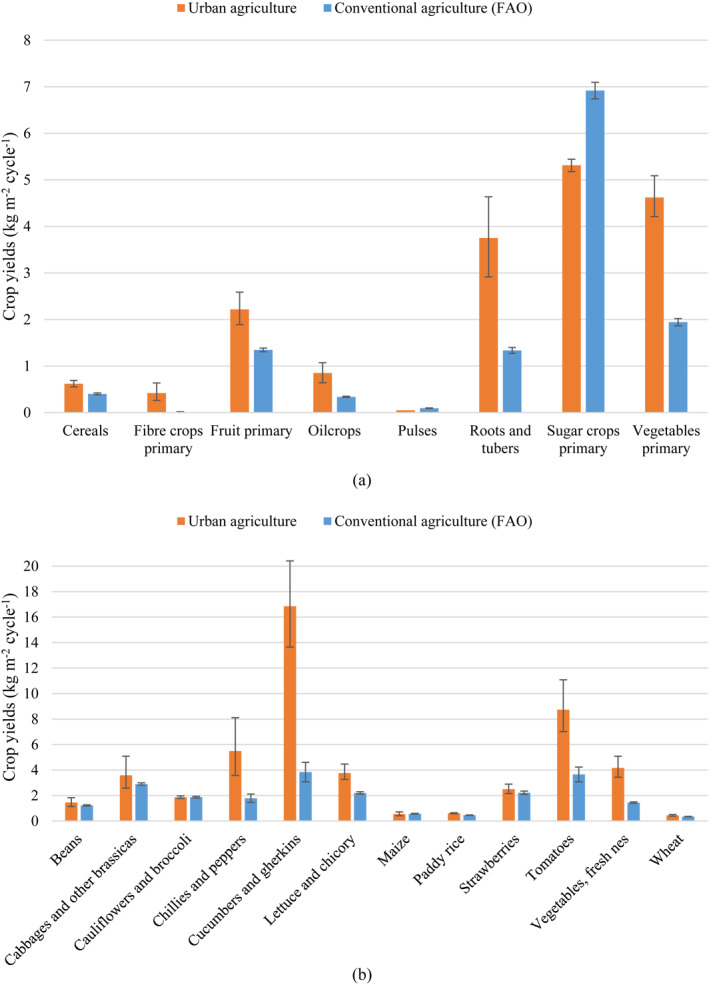
Mean crop yields per growing cycle of urban agriculture (data from this meta‐analysis) and mean global crop yields of conventional agriculture for the years 2015–2020 from FAOSTAT (FAO, 2022) for the aggregated (a) and disaggregated (b) crop categories. “Pulses” was not included in the analysis since only one observation was found for this crop category. Only disaggregated crop categories with a number of observations greater than 50 are shown. Bars represent non‐weighted mean yield values. Error bars correspond to the 95% confidence intervals.

The average crop yields of urban agriculture by aggregated and disaggregated crop category are presented in Table S1. These results were compared to the global yields of conventional agriculture published by the FAO (2022) for the years 2015–2020 (Figure 3). All the aggregated crop categories for which observations were found appear in Figure 3a; however, only the disaggregated crop categories with a number of observations greater than 50 were included in Figure 3b.

Regarding aggregated crop categories (Figure 3a), the strongest differences in yields were found for “fiber crops primary,” for which urban agricultural yields (0.42 kg m^−2^ cycle^−1^) were 42 times higher than conventional agricultural yields (0.010 kg m^−2^ cycle^−1^). Urban agricultural yields of “roots and tubers,” “oilcrops” and “vegetables primary” (3.8, 0.85, and 4.6 kg m^−2^ cycle^−1^, respectively) were more than twice as high as conventional yields (1.3, 0.34, and 1.9 kg m^−2^ cycle^−1^, respectively). Urban agricultural yields of “fruit primary” and “cereals” (2.2 and 0.62 kg m^−2^ cycle^−1^, respectively) were also higher than conventional ones (1.4 and 0.40 kg m^−2^ cycle^−1^, respectively), though to a lesser extent (1.6 times higher for both categories). “Sugar crops primary” was the only aggregated category for which urban agricultural yields (5.3 kg m^−2^ cycle^−1^) were lower than conventional yields (6.9 kg m^−2^ cycle^−1^).

Regarding disaggregated crop categories (Figure 3b), the strongest differences in yields were observed for “cucumbers and gherkins,” for which urban agricultural yields (17 kg m^−2^ cycle^−1^) were 4.4 times higher than conventional yields (3.8 kg m^−2^ cycle^−1^). The urban yields of the crop categories “tomatoes,” “vegetables, fresh nes” and “chillies and peppers” (8.7, 4.2, and 5.5 kg m^−2^ cycle^−1^, respectively) were between 2.4 and 3.1 times higher than conventional yields (3.7, 1.4, and 1.8 kg m^−2^ cycle^−1^, respectively). Urban yields of “lettuce and chicory” (3.8 kg m^−2^ cycle^−1^) were also higher than conventional yields (2.2 kg m^−2^ cycle^−1^), but to a lesser extent than for the previous categories of crops (only 1.7 times higher). The rest of the crop categories—“beans,” “cabbages and other brassicas,” “cauliflowers and broccoli,” “maize,” “paddy rice,” “strawberries,” and “wheat”—had similar urban (1.5, 3.6, 1.9, 0.55, 0.61, 2.5, and 0.44 kg m^−2^ cycle^−1^, respectively) and conventional yields (1.2, 2.9, 1.9, 0.57, 0.46, 2.2, and 0.35 kg m^−2^ cycle^−1^, respectively).

### 3.3 Influence of the Type of Urban Spaces on Urban Agricultural Yields

**Figure 4:**
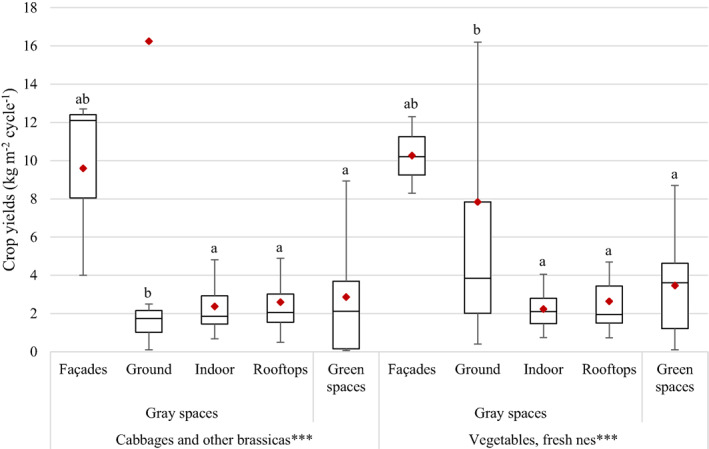
Differences in crop yields per growing cycle between urban spaces for two disaggregated categories of crops. Boxplots represent the first quartile (bottom end of the box), the median (band inside the box) and the third quartile (top end of the box). Error bars represent the minimum and maximum values of crop yields within the 1.5 interquartile range of the lower and upper quartiles, respectively. Red diamonds show the mean. *** = *p* < 0.001 (one‐way analysis of variance test). Absolutely different lower‐case letters represent a significant difference between categories, while there is no significant difference between categories with one same lower‐case letter (Tukey's honest significant difference test).

The differences in crop yields depending on the type of urban space are shown in Figure 4 for two disaggregated crop categories (for which there were available observations in each category and sub‐category of urban spaces). Overall, there is a significant effect of urban spaces on crop yields for both “cabbages and other brassicas” and “vegetables, fresh nes” (*p* = 0.000 for both crop categories). For “cabbages and other brassicas,” the yields of ground‐based urban spaces (16 kg m^−2^ cycle^−1^) were significantly higher than those of indoor spaces (2.4 kg m^−2^ cycle^−1^; *p* = 0.000), rooftops (2.6 kg m^−2^ cycle^−1^; *p* = 0.000) and green spaces (2.9 kg m^−2^ cycle^−1^; *p* = 0.000). There was no significant difference between the yields of the other urban spaces. For “vegetables, fresh nes,” the yields of ground‐based urban spaces (7.8 kg m^−2^ cycle^−1^) were also significantly higher than those of indoor spaces (2.2 kg m^−2^ cycle^−1^; *p* = 0.000), rooftops (2.6 kg m^−2^ cycle^−1^; *p* = 0.015) and green spaces (3.5 kg m^−2^ cycle^−1^; *p* = 0.001). Similarly, there was no significant difference between the yields of the other urban spaces. The categories “façades” for “cabbages and other brassicas,” “ground” for “vegetables, fresh nes,” and “green spaces” showed particularly high variability in the data. For “cabbages and other brassicas,” the category “ground” also exhibited a very positively skewed distribution of the data, with the value of the mean (16 kg m^−2^ cycle^−1^) being more than nine times higher than that of the median (1.7 kg m^−2^ cycle^−1^) for this category.

### 3.4 Influence of the Growing System on Urban Agricultural Yields

In the following sections, only the disaggregated crop categories with available data and enough yield values (i.e., > 5) in each category of the three aspects of the growing system considered were analyzed.

#### 3.4.1 Vertical versus Horizontal Farming

**Figure 5:**
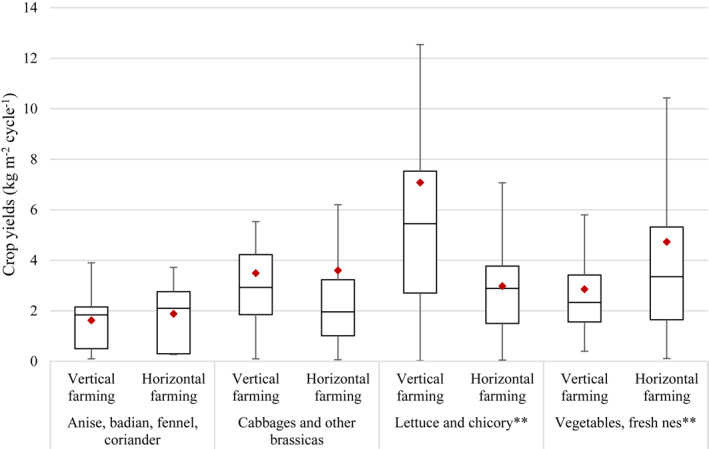
Differences in crop yields per growing cycle between urban systems using vertical farming and urban systems using horizontal farming for four disaggregated categories of crops. For vertical farming, crop yields correspond to the total weight of crops from all the different growing layers stacked together per square meter of ground area. Boxplots represent the first quartile (bottom end of the box), the median (band inside the box) and the third quartile (top end of the box). Error bars represent the minimum and maximum values of crop yields within the 1.5 interquartile range of the lower and upper quartiles, respectively. Red diamonds show the mean. ** = *p* < 0.01 (independent two‐sample *t*‐test).

Whether farming was conducted vertically or horizontally had a significant effect on crop yields for “lettuce and chicory” and “vegetables, fresh nes” (*p* = 0.006 and 0.005, respectively), but not for “anise, badian, fennel, coriander” nor for “cabbages and other brassicas” (*p* = 0.645 and 0.904, respectively). The effect, however, varied between crop categories: while vertical farming led to higher yields than horizontal farming in the case of “lettuce and chicory,” it was associated with lower yields than horizontal farming in the case of “vegetables, fresh nes” (Figure 5). For “lettuce and chicory,” yields of vertical farming (7.1 kg m^−2^ cycle^−1^) were, on average, 2.4 times higher than yields of horizontal farming (3.0 kg m^−2^ cycle^−1^), though the variability of the data in each category was very high (e.g., minimum yield value of vertical farming = 0.020 kg m^−2^ cycle^−1^ << maximum yield value of vertical farming = 13 kg m^−2^ cycle^−1^). For “vegetables, fresh nes,” mean yields of vertical farming (2.9 kg m^−2^ cycle^−1^) were 1.7 times lower than mean yields of horizontal farming (4.7 kg m^−2^ cycle^−1^), with also high variability in each category.

#### 3.4.2 Hydroponic versus Soil‐Based Systems

**Figure 6:**
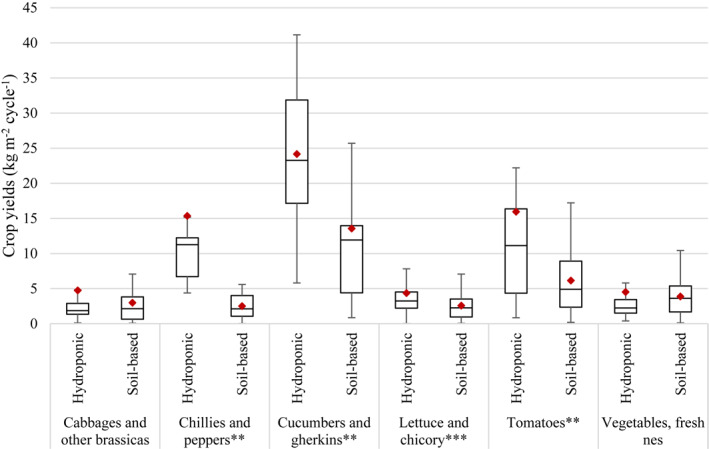
Differences in crop yields per growing cycle between hydroponic systems and soil‐based systems in urban environments for six disaggregated categories of crops. Boxplots represent the first quartile (bottom end of the box), the median (band inside the box) and the third quartile (top end of the box). Error bars represent the minimum and maximum values of crop yields within the 1.5 interquartile range of the lower and upper quartiles, respectively. Red diamonds show the mean. ** = *p* < 0.01; *** = *p* < 0.001 (independent two‐sample *t*‐test).

While the growing medium had a significant effect on crop yields for “chillies and peppers,” “cucumbers and gherkins,” “lettuce and chicory,” and “tomatoes” (*p* = 0.009, 0.002, 0.000, and 0.009, respectively), its effect was not significant for “cabbages and other brassicas” nor for “vegetables, fresh nes” (*p* = 0.364 and 0.500, respectively). Hydroponic systems were associated with higher average yields than soil‐based systems for all the crop categories where the effect of the type of medium used to grow the crop was significant (Figure 6). The differences in average yields between hydroponic and soil‐based systems were high (i.e., > 2.5 times higher) for “tomatoes” (16 vs. 6.1 kg m^−2^ cycle^−1^, respectively) and very high (i.e., > 6 times higher) for “chillies and peppers” (15 vs. 2.5 kg m^−2^ cycle^−1^, respectively). This was less the case for “cucumbers and gherkins” and “lettuce and chicory,” for which the variation in yields between hydroponic and soil‐based systems was much lower (i.e., < twice higher). Data also showed very high variability within categories, particularly for “cucumbers and gherkins” and “tomatoes” (Figure 6).

#### 3.4.3 Controlled‐Environment versus Open‐Air Agriculture

**Figure 7:**
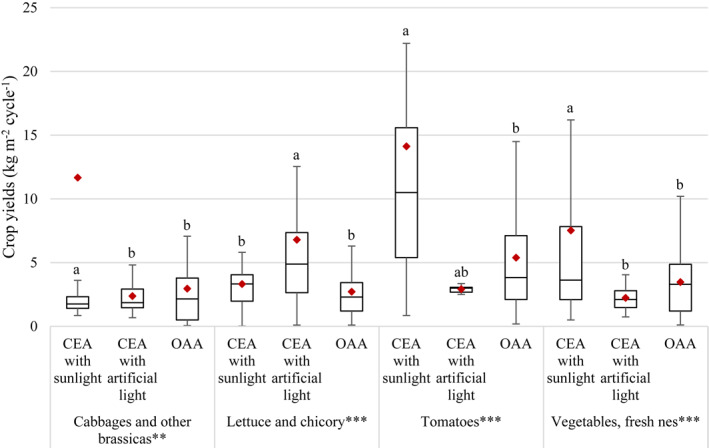
Differences in crop yields per growing cycle between controlled‐environment agriculture (CEA) and open‐air agriculture (OAA) for four disaggregated categories of crops. Boxplots represent the first quartile (bottom end of the box), the median (band inside the box) and the third quartile (top end of the box). Error bars represent the minimum and maximum values of crop yields within the 1.5 interquartile range of the lower and upper quartiles, respectively. Red diamonds show the mean. ** = *p* < 0.01; *** = *p* < 0.001 (one‐way analysis of variance test). Absolutely different lower‐case letters represent a significant difference between categories, while there is no significant difference between categories with one same lower‐case letter (Tukey's honest significant difference test).

The influence of the level of conditioning of the growing system on urban agricultural yields is shown in Figure 7 for four disaggregated crop categories. The level of conditioning of the growing system had a significant effect on crop yields for all crop categories (*p* = 0.001 for “cabbages and other brassicas” and *p* = 0.000 for “lettuce and chicory,” “tomatoes,” and “vegetables, fresh nes”). For “cabbages and other brassicas,” the yields of controlled‐environment agriculture with sunlight (12 kg m^−2^ cycle^−1^) were significantly higher than the yields of controlled‐environment agriculture with artificial light (2.4 kg m^−2^ cycle^−1^; *p* = 0.001) and open‐air agriculture (3.0 kg m^−2^ cycle^−1^; *p* = 0.001). There was no significant difference in yields between controlled‐environment agriculture with artificial light and open‐air agriculture. For “lettuce and chicory,” the yields of controlled‐environment agriculture with artificial light (6.8 kg m^−2^ cycle^−1^) were significantly higher than those of controlled‐environment agriculture with sunlight (3.3 kg m^−2^ cycle^−1^; *p* = 0.000) and open‐air agriculture (2.7 kg m^−2^ cycle^−1^; *p* = 0.000). The differences in yields observed between controlled‐environment agriculture with sunlight and open‐air agriculture were not significant for this category of crops. For “tomatoes,” the yields of controlled‐environment agriculture with sunlight (14 kg m^−2^ cycle^−1^) were significantly higher than those of open‐air agriculture (5.4 kg m^−2^ cycle^−1^; *p* = 0.000). The rest of the categories had no significantly different mean yields for this crop. For “vegetables, fresh nes,” the yields of controlled‐environment agriculture with sunlight (7.5 kg m^−2^ cycle^−1^) were significantly higher than those of controlled‐environment agriculture with artificial light (2.2 kg m^−2^ cycle^−1^; *p* = 0.000) and open‐air agriculture (3.5 kg m^−2^ cycle^−1^; *p* = 0.000). There was no significant difference in yields between controlled‐environment agriculture with artificial light and open‐air agriculture. There was high variability in the data, especially in the case of “controlled‐environment agriculture with sunlight” and “open‐air agriculture” for both “tomatoes” and “vegetables, fresh nes.”

## 4 Discussion

### 4.1 Crop Yields of Urban Agriculture

To our knowledge, Dorr et al. (2021) is the only other study reporting crop yields of urban agriculture by way of meta‐analysis. As crop yields were reported per year instead of per growing cycle in their study, we recalculated the crop yields in our meta‐analysis per year instead of per growing cycle so our results would be comparable. We also combined some of the crop categories used in Dorr et al. (2021)'s meta‐analysis so they would match our disaggregated crop categories (e.g., “tomato” and “tomato (cherry)” were combined to become “tomatoes” while “lettuce” and “chicory” were merged into “lettuce and chicory”). We found that the values of urban agricultural yields estimated in our study varied considerably from those reported by Dorr et al. (2021). Focusing on “lettuce and chicory” and “tomatoes” only, crop yields found in our meta‐analysis were notably lower than those calculated by Dorr et al. (2021): 5.5 versus 19 kg m^−2^ yr^−1^ for “lettuce and chicory” and 8.7 versus 15 kg m^−2^ yr^−1^ for “tomatoes,” respectively. These differences in productivity of urban agriculture could be explained by the variation in the number of observations considered to estimate crop yields, which was much higher in our study (*n* = 344 vs. 41 for “lettuce and chicory” and 208 vs. 29 for “tomatoes”). The higher occurrence of growing systems with several growing cycles a year (sometimes up to 12) in the data set used in Dorr et al. (2021) could also be responsible for the differences in yearly crop yields observed. Finally, these differences could be due to the specific inclusion criteria used in the literature search of our meta‐analysis, according to which studies that extrapolated urban yields from conventional yields or used yields estimated via modeling were excluded.

Other studies focusing on the food production potential at the city level (e.g., Gondhalekar & Ramsauer, 2017; Hsieh et al., 2017; Saha & Eckelman, 2017) have also reported crop yields of urban agriculture and used them in their assessments of the extent to which urban food production may lead to self‐sufficiency in cities and towns. The crop yields estimated in these studies differed considerably from those calculated in our meta‐analysis. For instance, crop yields of dark green vegetables used by Saha and Eckelman (2017) were lower than those estimated in our meta‐analysis for conventional urban gardening (1.4 vs. 2.6 kg m^−2^ yr^−1^, respectively) but much greater than the values from our meta‐analysis for hydroponic rooftop gardening (20 vs. 8.6 kg m^−2^ yr^−1^, respectively). These differences in yields may be due to the fact that these scaling‐up studies extrapolated yield values from specific studies based on local field experiments (Saha & Eckelman, 2017), from particular agri‐businesses (Hsieh et al., 2017), or from conventional agriculture (Gondhalekar & Ramsauer, 2017). This suggests that these studies may have underestimated or overestimated the productive potential of the cities and towns that they considered due to their reliance on proxy or extrapolated data instead of self‐determined or commercially established figures. In their review of existing self‐sufficiency studies relating to urban agriculture, Weidner et al. (2019) observed that only a few studies overall employed thoroughly evaluated yield figures such as yield data from commercial projects, realistic simulations or ongoing research in their calculations, which negatively impacted the accuracy of their estimates of how self‐sufficient cities and towns can be in terms of food production. This highlights how crucial global empirical data of crop yields in urban agriculture such as those compiled in this meta‐analysis are to accurately estimate the productive potential of urban areas.

### 4.2 Is Urban Agriculture More Productive Than Conventional Agriculture?

Results from this meta‐analysis show that urban agriculture has, overall, a high potential for food production and that urban spaces can be as productive or even more productive than rural environments, with mean crop yields of urban agriculture being similar to or greater than conventional agricultural yields (Figure 3). This is consistent with the results of Dorr et al. (2021), who, in their meta‐analysis, also found urban agricultural yields to be on par with or, at times, much greater than conventional agricultural yields, and of McDougall et al. (2019), who found that the productivity of urban agriculture in Australia exceeded that of rural farms for vegetable crops. It also corroborates the findings of previous studies suggesting that the productivity of urban agriculture was improved over that of conventional agriculture (Despommier, 2013; Goldstein et al., 2016).

The differences in yields observed between urban and conventional agriculture in our study might be due to the high occurrence of crops grown in controlled environments in urban agriculture (42% of our data set corresponded to crops grown in such environments), where crops are shielded from moderating variables (such as extreme weather, pests and diseases), though controlled environments also occur in conventional agriculture (Goldstein et al., 2016). Another explanation could be the presence of vertical farming in urban areas, which substantially increases the amount of food produced per area, while also offering controlled growing conditions, often using hydroponics, artificial lighting, controlled temperature, etc. (Avgoustaki & Xydis, 2020a). 10% of the data analyzed in our study took place in vertical systems. Ultimately, the higher productivity of urban agriculture may also be linked to the substitution of mechanical labor with mostly manual labor in urban food production, which allows for a higher cropping density than in machine‐managed systems (Morel et al., 2017).

### 4.3 Which Urban System Has the Highest Food Production Potential?

To avoid skewing the effect of urban spaces and growing systems on crop yields, the data set was split and analyses were conducted by crop category. As a result, we were unable to observe an overall trend regarding how food productivity varied based on where and how crops were grown. Nevertheless, the analyses of several crops showed that specific urban spaces and growing systems led to higher crop yields than others.

It was surprising to find no significant difference between most urban spaces, and especially between indoor and green spaces. Indoor spaces corresponded exclusively to controlled‐environment systems with artificial light and the use of hydroponics, while green spaces referred mostly to soil‐based, open‐air systems (though a few used polytunnels). Controlled‐environment agriculture combined with the use of hydroponics creates optimized conditions for crop growth, leading to maximized crop production and yields due to precise application of inputs, controlled growth parameters and reduced exposure to pests and diseases (Ragaveena et al., 2021). Soilless agriculture also helps to reduce nutritional deficiency in crops, maintain soil pH via nutrient solution dosage and avoid problems traditionally linked to soil‐based production such as pests and diseases (Li et al., 2020). Yet, in the case of urban agriculture, indoor spaces did not impact crop yields differently than green spaces for “cabbages and other brassicas” nor for “vegetables, fresh nes,” even though controlled‐environment agriculture led to significantly higher yields than open‐air agriculture for these crop categories. This may partly be due to the higher variability in the data for green spaces than for non‐façade gray spaces and to the fact that “ground” samples were very positively skewed by some results with very high yields, which might have caused the statistical methods used on this category to be misleading.

It is worth noting that differences in productivity between growing systems are not only system‐dependent but also crop‐dependent. Several disaggregated crop categories used in this meta‐analysis included different types of crops with varying suitability to specific urban spaces or growing systems. For instance, although the yields of “ground” spaces were significantly higher than those of “indoor” spaces for “cabbages and other brassicas,” the types of brassicas used in the two types of spaces were very different, despite them being gathered into the same crop category: for indoor spaces, it was mainly leafy brassicas (e.g., mustard greens), while ground‐based spaces welcomed heavier types of brassicas (e.g., cabbages). The same was true for the category “vegetables, fresh nes,” which encompassed very diverse kinds of vegetables. Moreover, the differences in yields between vertical and horizontal farming for “vegetables, fresh nes” might be due to the distinct types of vegetables grown in each system (e.g., beet salad leaves in vertical systems vs. beetroots in horizontal systems) whose yields intrinsically are in different orders of magnitude. This might explain why vertical farming was associated with lower yields than horizontal farming for this crop category, which was unexpected due to the much higher density of crops in vertical systems. However, despite the limitations of some FAOSTAT categories, especially those that include different types of crops (in terms of weight or even nature) and those that separate crops that should have been gathered together (e.g., cauliflowers and broccoli being apart from “cabbages and other brassicas”), choosing corresponding categories in this meta‐analysis allowed us to establish comparisons with the global yield values from the FAOSTAT database, which provides the most robust benchmark for the productivity of conventional agriculture. Though more suited categories of crops could be defined in further studies relating to the productivity of urban agriculture, the lack of global data on the yields of conventional agriculture outside of the FAOSTAT database may hinder the extent to which these future results could be compared to conventional agriculture.

Using results from this meta‐analysis, we can seek to draw the characteristics of the best‐performing systems for optimized crop production in urban environments. If we take the example of “tomatoes,” yields were the highest for hydroponic greenhouses (i.e., systems using horizontal farming, hydroponic methods and a controlled environment with sunlight). Based on our data set, such systems deliver average yields of 18 kg m^−2^ cycle^−1^ for tomatoes, which is more than thrice higher (*p* = 0.004) than tomato yields achieved in urban soil‐based, open‐air green spaces (5.3 kg m^−2^ cycle^−1^).

Another example would be that of “lettuce and chicory,” for which yields were the highest in systems using vertical farming, hydroponic methods and a controlled environment with artificial light (such as plant factories, growth chambers or repurposed shipping containers). Based on our data set, such systems achieve average yields of 17 kg m^−2^ cycle^−1^ for lettuces and chicories, which is more than six times higher (*p* = 0.004) than yields obtained in urban soil‐based, open‐air green spaces (2.8 kg m^−2^ cycle^−1^). Also, because these elaborate systems may, on average, grow fully mature lettuces and chicories in a month and controlled environments enable lettuce and chicory production all year round, it is estimated that 12 growing cycles of lettuces and chicories a year could be successfully achieved in plant factories (Pennisi et al., 2019; Su et al., 2020). This could bring the average yields of “lettuce and chicory” up to 204 kg m^−2^ year^−1^, assuming yields of 17 kg m^−2^ cycle^−1^. By comparison, green spaces could accommodate much fewer cycles during the growing season of lettuces and chicories. However, achieving these yields using plant factories may be hindered by the investment and operating costs associated with such structures, which tend to be high in many urban settings (Thomaier et al., 2015). Plant factories also require more technology development and are associated with higher energy use than conventional systems (Kikuchi et al., 2018).

### 4.4 Uncertainty and Further Research

Despite the broad scope of our literature search, there were important variations in the number of observations gathered for each of the categories of crops created in the study. Certain crops had too few observations for them to be analyzed properly and had to be excluded from the analysis, while others did not have enough observations to investigate potential differences among urban spaces or growing systems. This highlights the fact that the literature on the productivity of urban agriculture tends to focus on particular crops (such as leafy greens and tomatoes) and overlook others (mainly fruit)—studies specifically addressing soft fruit and orchard fruit were an unexpected gap given the likely prevalence of these crops in urban areas. Studies were also sparse for specific crop categories in gray spaces. For instance, though there is a considerable rise in interest in the use of green walls, the number of observations for this urban space was still very low (*n* = 11) and limited to certain crops (such as lettuces, tomatoes, cooking herbs and brassicas). It was surprising to notice, however, that cereals, which are mostly seen as crops better suited to rural environments, were relatively well studied in the literature on urban agriculture, and that their agricultural productivity was even higher in urban settings.

Even for the crop categories with a number of observations high enough for them to be included in the analysis, there were important differences in the number of yield values used to estimate crop yields between crop categories. For example, the estimated crop yields for “vegetables primary” were based on 1,569 observations, while those of “fiber crops primary” and “sugar crops primary” were based on only 13 and 8 observations, respectively. The same remark can be made between the yields of “lettuce and chicory” (based on 344 observations) and those of “cucumbers and gherkins” (based on 58 observations). This variation in the number of observations might be a limitation to how representative or generalizable the findings might be depending on the crops considered. In addition, the estimates of crop yields quantified in this paper were based on the observations found for each crop category. However, the types of growing systems for each crop category varied: for some crop categories, the average urban agricultural yields estimated stemmed more from open‐air systems (e.g., 91% and 81% of the yield values observed for “maize” and “wheat” were from open‐air systems, respectively) while, for others, they resulted more from controlled‐environment systems (e.g., 81% and 60% of the yield values observed for “cucumbers and gherkins” and “lettuce and chicory” were from controlled‐environment systems, respectively). This may partly explain why certain crop categories such as “cucumbers and gherkins” and “lettuce and chicory” had such higher crop yields for urban agriculture than for conventional farming while others did not. This was not the case, however, for all the crop categories with a prevalence of controlled‐environment systems. For example, 92% and 84% of the yield values for “cauliflowers and broccoli” and “strawberries” in our data set were from controlled‐environment systems; yet, the average yields of these crop categories for urban agriculture were similar to those for conventional agriculture.

Another element that potentially affected observed crop yields is the fact that a high number of observations in our sample derived from field experiments set up and led by researchers, who planted, grew and harvested the crops themselves. In such experiments, there is a high level of control and precision given to the crop—inputs are carefully applied and measured, crop growth monitored, and pests and diseases controlled. However, this differs to some extent from the reality of crops grown by individuals or farmers in urban settings, and it may lead to increased yields when compared to real‐life scenarios. There is, therefore, a need for more studies that report yields from existing urban farms, such as the Brooklyn Grange farm in New York City (Brooklyn Grange Farm, 2022), or use citizens science experiments, such as the MYHarvest initiative in the United Kingdom (Edmondson et al., 2022), rather than experiments undertaken by researchers. The former approach is more representative of actual conditions under which urban agriculture is conducted in cities and towns.

This study provides underpinning systematic evidence that can be used in developing a clearer picture of the extent to which urban food growing could help to meet global food demand, reduce food insecurity (by increasing food availability and access to food) and make cities and towns (and by extension the whole food system) more resilient to shocks. However, there is still uncertainty regarding the sustainability of urban food production and how it may vary depending on where and how crops are grown (McDougall et al., 2019). If urban agriculture happens to be more resource‐intensive (in particular regarding energy, water, nutrient consumption and waste disposal) than conventional agriculture, this could represent a barrier to the deployment of urban agriculture at a large scale. The same reasoning applies to different urban spaces: in an analysis comparing the environmental impact and resource use of Japanese horticultural systems, Kikuchi et al. (2018) found that plant factories led to higher energy consumption than conventional systems, even though they reduced phosphorus, water and land requirements for food production. Further life cycle assessments of urban agriculture need to be conducted to assess whether producing food in cities and towns for urban dwellers is more resource‐efficient than existing supply chains. This would consolidate the design and management of urban agricultural systems (Dorr et al., 2021) and help to deliver more accurate estimates of the efficiency and performance of urban spaces at producing sufficient yields to meet demand in the most sustainable way (Avgoustaki & Xydis, 2020b).

## 5 Conclusions

The main objective of this meta‐analysis was to assess the agricultural productivity of different urban systems, and how it compares with that of conventional agriculture. We found a broad range of crops being grown in urban spaces, mostly vegetables, but also cereals and fruit. “Lettuce and chicory” was the most studied crop category. The capacity of urban areas to produce food was strong, with urban agricultural yields largely on par with or higher than conventional yields. A wide variety of urban spaces were also found in the literature search, with variations depending on their location (ground‐based, roof‐based, indoor, etc.) or production system (hydroponic or soil‐based, vertical or horizontal, controlled‐environment or open‐air). Their effect on crop yields was analyzed for a series of crop categories, though it was not significant for all. Ground‐based spaces had significantly higher crop yields than indoor spaces, rooftops and green spaces for “cabbages and other brassicas” and “vegetables, fresh nes.” The effect on yields of whether crops were grown vertically or horizontally was significant for “lettuce and chicory” and “vegetables, fresh nes,” but the impact varied between the two crop categories—vertical farming led to higher yields than horizontal farming in the case of “lettuce and chicory” but to lower yields for “vegetables, fresh nes.” Hydroponic systems were associated with higher yields than soil‐based systems for “chillies and peppers,” “cucumbers and gherkins,” “lettuce and chicory,” and “tomatoes.” Crop yields of controlled‐environment agriculture were significantly higher than those of open‐air agriculture for several crops (“cabbages and other brassicas,” “lettuce and chicory,” “tomatoes,” and “vegetables, fresh nes”). Overall, our meta‐analysis represents the first attempt at quantifying the globally relevant crop yields of urban food growing. Results from this study contribute to building a more robust evidence base for urban agriculture. They also provide valuable resources for more accurate scaling‐up research seeking to estimate the agricultural productivity and self‐sufficiency of cities and towns across the globe and for further life cycle assessments of urban agriculture, which could contribute to designing urban production systems that support a more sustainable future.

## Supporting information

Supporting Information S1

Table S1

Data Set S1

## Data Availability

Data used in this research have been extracted from the 200 studies listed in Supporting Information S1. A combined data set summarizing the extracted data is available in Data Set S1.
