# Supplementary material for: How Much Food Can We Grow in Urban Areas? Food Production and Crop Yields of Urban Agriculture: A Meta‐Analysis
Source: Earths Future. 2022 Aug 26;10(8):e2022EF002748. doi: 10.1029/2022EF002748 (PMC9540868; doi:10.1029/2022EF002748)
Supplement: Supplementary file 1 — Supporting Information S1 [file EFT2-10-e2022EF002748-s001.docx]

**Supporting Information S1.** Studies included in the meta-analysis.

Adamtey, N., Cofie, O., Ofosu-Budu, K. G., Ofosu-Anim, J., Laryea, K. B., & Forster, D. (2010). Effect of N-enriched co-compost on transpiration efficiency and water-use efficiency of maize (Zea mays L.) under controlled irrigation. *Agricultural Water Management*, *97*(7), 995–1005. https://doi.org/10.1016/j.agwat.2010.02.004

Affokpon, A., Coyne, D. L., Htay, C. C., Dossou Agbèdè, R., Lawouin, L., & Coosemans, J. (2011). Biocontrol potential of native Trichoderma isolates against root-knot nematodes in West African vegetable production systems. *Soil Biology & Biochemistry*, *43*(3), 600–608. https://doi.org/10.1016/j.soilbio.2010.11.029

Agrawal, M., Singh, B., Rajput, M., Marshall, F., & Bell, J. N. B. (2003). Effect of air pollution on peri-urban agriculture: a case study. *Environmental Pollution*, *126*(3), 323–329. https://doi.org/10.1016/S0269-7491(03)00245-8

Akoto-Danso, E. K., Manka’abusi, D., Steiner, C., Werner, S., Häring, V., Nyarko, G., et al. (2019). Agronomic effects of biochar and wastewater irrigation in urban crop production of Tamale, northern Ghana. *Nutrient Cycling in Agroecosystems*, *115*, 231–247. https://doi.org/10.1007/s10705-018-9926-6

Al-Far, A. M., Tadros, M. J., & Makhadmeh, I. M. (2019). Evaluation of different soilless media on growth, quality, and yield of cucumber (Cucumis sativus L.) grown under greenhouse conditions. *Australian Journal of Crop Science*, *13*(8), 1388–1400. https://doi.org/10.21475/ajcs.19.13.08.p2122

Aliyu, B., & Wachap, E. (2014). Vegetable cowpea as a source of cheap protein and an environmentally friendly crop for urban cities. *WIT Transactions on Ecology and the Environment*, *181*, 301–312. https://doi.org/10.2495/EID140261

Al-Mulla, Y. A., Al-Busaidi, H., & Al-Balushi, M. (2014). Soilless culture in controlled environment: Mechanisms of recovering crop production of the salt affected lands due to seawater intrusion. In *2014 ASABE – CSBE/SCGAB Annual International Meeting Paper* (pp. 1–8). Montreal, Quebec, Canada: American Society of Agricultural and Biological Engineers. https://doi.org/10.13031/aim.20141913540

Alomran, A. M., Louki, I. I., Aly, A. A., & Nadeem, M. E. (2013). Impact of deficit irrigation on soil salinity and cucumber yield under greenhouse condition in an arid environment. *Journal of Agricultural Science and Technology*, *15*(6), 1247–1259. https://doi.org/10.2495/SI140111

Alu’datt, M. H., Rababah, T., Alhamad, M. N., Al-Tawaha, A., Al-Tawaha, A. R., Gammoh, S., et al. (2019). Herbal yield, nutritive composition, phenolic contents and antioxidant activity of purslane (Portulaca oleracea L.) grown in different soilless media in a closed system. *Industrial Crops & Products*, *141*, 1–8. https://doi.org/10.1016/j.indcrop.2019.111746

Amoah, P., Adamtey, N., & Cofie, O. (2017). Effect of urine, poultry manure, and dewatered faecal sludge on agronomic characteristics of cabbage in Accra, Ghana. *Resources*, *6*(2), 1–14. https://doi.org/10.3390/resources6020019

Asseng, S., Guarin, J. R., Raman, M., Monje, O., Kiss, G., Despommier, D. D., et al. (2020). Wheat yield potential in controlled-environment vertical farms. *Proceedings of the National Academy of Sciences of the United States of America*, *117*(32), 19131–19135. https://doi.org/10.1073/pnas.2002655117

Bączek, K., Kosakowska, O., Gniewosz, M., Gientka, I., & Węglarz, Z. (2019). Sweet basil (Ocimum basilicum L.) productivity and raw material quality from organic cultivation. *Agronomy*, *9*(6), 1–15. https://doi.org/10.3390/agronomy9060279

Baganz, G., Baganz, D., Staaks, G., Monsees, H., & Kloas, W. (2020). Profitability of multi-loop aquaponics: Year-long production data, economic scenarios and a comprehensive model case. *Aquaculture Research*, *51*(7), 2711–2724. https://doi.org/10.1111/are.14610

Bantis, F., Fotelli, M., Ilić, Z. S., & Koukounaras, A. (2020). Physiological and phytochemical responses of spinach baby leaves grown in a PFAL system with LEDs and saline nutrient solution. *Agriculture*, *10*(11), 1–12. https://doi.org/10.3390/agriculture10110574

Bassegio, D., & Dutra Zanotto, M. (2020). Growth, yield, and oil content of Brassica species under Brazilian tropical conditions. *Bragantia*, *79*(2), 203–212. https://doi.org/10.1590/1678-4499.20190411

Belhachemi, A., Maatoug, M., Amirat, M., & Dehbi, A. (2020). A study of the growth and yield of Solanum lycopersicum under greenhouses differentiated by the LDPE cover-film. *Ukrainian Journal of Ecology*, *10*(2), 69–75. https://doi.org/10.15421/2020_66

Beniston, J. W., Lal, R., & Mercer, K. L. (2016). Assessing and managing soil quality for urban agriculture in a degraded vacant lot soil. *Land Degradation and Development*, *27*(4), 996–1006. https://doi.org/10.1002/ldr.2342

Boneta, A., Rufí-Salís, M., Ercilla-Montserrat, M., Gabarrell, X., & Rieradevall, J. (2019). Agronomic and environmental assessment of a polyculture rooftop soilless urban home garden in a Mediterranean city. *Frontiers in Plant Science*, *10*(341), 1–11. https://doi.org/10.3389/fpls.2019.00341

Bosc, J.-P., & Grisey, A. (2019). Effect of growing strawberries with superimposed levels of cropgutters on yield and quality. *European Journal of Horticultural Science*, *84*(1), 24–30. https://doi.org/10.17660/eJHS.2019/84.1.4

Bouchaaba, Z., Santamaria, P., Choukr-Allah, R., Lamaddalena, N., & Montesano, F. F. (2015). Open-cycle drip vs closed-cycle subirrigation: Effects on growth and yield of greenhouse soilless green bean. *Scientia Horticulturae*, *182*, 77–85. https://doi.org/10.1016/j.scienta.2014.11.007

Busari, I. T., Senzanje, A., Odindo, A. O., & Buckley, C. A. (2019). The impact of irrigation water management techniques on the performance of rice using treated wastewater reuse in Durban, South Africa. *Water Supply*, *19*(6), 1604–1611. https://doi.org/10.2166/ws.2019.031

Bustamante, N., Acuña, J. F., & Valera, D. L. (2015). Effect of the height of the greenhouse on the plant - climate relationship as a development parameter in mint (Mentha Spicata) crops in Colombia. *Ingeniería e Investigación*, *36*(2), 6–13. https://doi.org/10.15446/ing.investig.v36n2.52775

Calero Hurtado, A., Pérez Díaz, Y., Peña Calzada, K., Quintero Rodríguez, E., & Olivera Viciedo, D. (2019). Effect of three bio-stimulants in the morphologic and productive behavior of radish crops (Raphanus sativus L.). *Revista de La Facultad de Agronomía*, *36*(1), 54–73.

Caputo, P., Zagarella, F., Cusenza, M. A., Mistretta, M., & Cellura, M. (2020). Energy-environmental assessment of the UIA-OpenAgri case study as urban regeneration project through agriculture. *Science of the Total Environment*, *729*, 1–14. https://doi.org/10.1016/j.scitotenv.2020.138819

Carotti, L., Graamans, L., Puksic, F., Butturini, M., Meinen, E., Heuvelink, E., & Stanghellini, C. (2021). Plant factories are heating up: Hunting for the best combination of light intensity, air temperature and root-zone temperature in lettuce production. *Frontiers in Plant Science*, *11*, 1–11. https://doi.org/10.3389/fpls.2020.592171

Castoldi da Costa, R., Caglioni Durante, G., dos Santos Trentin, N., Trevizan Chiomento, J. L., Stockmans de Nardi, F., Nienow, A. A., & Oliveira Calvete, E. (2020). Micorrhizal biotechnology as an alternative to potentialize the strawberry quality. *Bioscience Journal*, *36*(5), 1619–1628. https://doi.org/10.14393/BJ-v36n5a2020-42816

CoDyre, M., Fraser, E. D. G., & Landman, K. (2015). How does your garden grow? An empirical evaluation of the costs and potential of urban gardening. *Urban Forestry & Urban Greening*, *14*(1), 72–79. https://doi.org/10.1016/j.ufug.2014.11.001

Contreras, J. I., Eymar, E., Lopez, J. G., Lao, M. T., & Segura, M. L. (2013). Influences of nitrogen and potassium fertigation on nutrient uptake, production, and quality of pepper irrigated with disinfected urban wastewater. *Communications in Soil Science and Plant Analysis*, *44*(1–4), 767–775. https://doi.org/10.1080/00103624.2013.748858

Dalla Marta, A., Baldi, A., Lenzi, A., Lupia, F., Pulighe, G., Santini, E., et al. (2019). A methodological approach for assessing the impact of urban agriculture on water resources: a case study for community gardens in Rome (Italy). *Agroecology and Sustainable Food Systems*, *43*(2), 228–240. https://doi.org/10.1080/21683565.2018.1537323

Dao, J., Lompo, D. J.-P., Stenchly, K., Haering, V., Marschner, B., & Buerkert, A. (2019). Gypsum amendment to soil and plants affected by sodic alkaline industrial wastewater irrigation in urban agriculture of Ouagadougou, Burkina Faso. *Water, Air, & Soil Pollution*, *230*, 1–12. https://doi.org/10.1007/s11270-019-4311-x

de la Caridad González Gort, D., Valido Tomes, A., Vázquez Montes de Oca, R., & Martínez González, H. (2018). Evaluation of effect of human urine fertilizer in the cultivation of corn in Camagüey. *Centro Agrícola*, *45*(4), 59–67. Retrieved from http://scielo.sld.cu/scielo.php?script=sci_abstract&pid=S0253-57852018000400059&lng=pt&nrm=iso

de los Milagros Orberá Ratón, T., Nápoles Vinent, S., de Jesús Serrat Díaz, M., Ortega Delgado, E., & Ramos Barbosa, H. (2014). The new rhizospheric bacteria Brevibacillus benefits eggplant and pepper growth and productivity under organoponic system. *Agricultural Research*, *3*(4), 395–398. https://doi.org/10.1007/s40003-014-0136-4

Demling, F. (2018). Food production on greening of roofs and façades. *Acta Horticulturae*, *1215*(32), 171–174. https://doi.org/10.17660/ActaHortic.2018.1215.32

Depardieu, C., Prémont, V., Boily, C., & Caron, J. (2016). Sawdust and bark-based substrates for soilless strawberry production: Irrigation and electrical conductivity management. *PLoS ONE*, *11*(4), 1–20. https://doi.org/10.1371/journal.pone.0154104

Diogo, R. V. C., Buerkert, A., & Schlecht, E. (2011). Economic benefit to gardeners and retailers from cultivating and marketing vegetables in Niamey, Niger. *Outlook on Agriculture*, *40*(1), 71–78. https://doi.org/10.5367/oa.2011.0027

Djidonou, D., & Leskovar, D. I. (2019). Seasonal changes in growth, nitrogen nutrition, and yield of hydroponic lettuce. *HortScience*, *54*(1), 76–85. https://doi.org/10.21273/HORTSCI13567-18

Dong, C., Shao, L., Fu, Y., Wang, M., Xie, B., Yu, J., & Liu, H. (2015). Evaluation of wheat growth, morphological characteristics, biomass yield and quality in Lunar Palace-1, plant factory, green house and field systems. *Acta Astronautica*, *111*, 102–109. https://doi.org/10.1016/j.actaastro.2015.02.021

Dorr, E., Sanyé-Mengual, E., Gabrielle, B., Grard, B. J.-P., & Aubry, C. (2017). Proper selection of substrates and crops enhances the sustainability of Paris rooftop garden. *Agronomy for Sustainable Development*, *37*(5), 1–11. https://doi.org/10.1007/s13593-017-0459-1

Ekoungoulou, R., & Mikouendanandi, E. B. R. M. (2020). Lettuce (Lactuca sativa L.) production in Republic of Congo using hydroponic system. *Open Access Library Journal*, *7*(5), 1–17. https://doi.org/10.4236/oalib.1106339

Endut, A., Jusoh, A., Ali, N., & Wan Nik, W. B. (2011). Nutrient removal from aquaculture wastewater by vegetable production in aquaponics recirculation system. *Desalination and Water Treatment*, *32*(1–3), 422–430. https://doi.org/10.5004/dwt.2011.2761

Engindeniz, S. (2004). The economic analysis of growing greenhouse cucumber with soilless culture system: The case of Turkey. *Journal of Sustainable Agriculture*, *23*(3), 5–19. https://doi.org/10.1300/J064v23n03_03

Engindeniz, S., & Gül, A. (2009). Economic analysis of soilless and soil-based greenhouse cucumber production in Turkey. *Scientia Agricola*, *66*(5), 606–614. https://doi.org/10.1590/s0103-90162009000500004

Fecondini, M., Casati, M., Dimech, M., Michelon, N., Orsini, F., & Gianquinto, G. (2009). Improved cultivation of lettuce with a low cost soilless system in indigent areas of Northeast Brazil. *Acta Horticulturae*, *807*(73), 501–508. https://doi.org/10.17660/ActaHortic.2009.807.73

Fernández-Cabanás, V. M., Pérez-Urrestarazu, L., Juárez, A., Kaufman, N. T., & Gross, J. A. (2020). Comparative analysis of horizontal and vertical decoupled aquaponic systems for basil production and effect of light supplementation by LED. *Agronomy*, *10*(9), 1–14. https://doi.org/10.3390/agronomy10091414

Ferreira de Oliveira Neto, C., Shigueru Okumura, R., de Jesus Matos Viégas, I., Oliveira da Conceição, H. E., Fragoso Monfort, L. E., Lima da Silva, R. T., et al. (2014). Effect of water stress on yield components of sorghum (Sorghum bicolor). *Journal of Food, Agriculture & Environment*, *12*(3–4), 223–228.

García Lozano, M., Escobar, I., & Berenguer, J. J. (2005). Green-pepper fertigation in soilless culture. *Acta Horticulturae*, *697*(71), 543–547. https://doi.org/10.17660/ActaHortic.2005.697.71

García-Delgado, C., Eymar, E., Contreras, J. I., & Segura, M. L. (2012). Effects of fertigation with purified urban wastewater on soil and pepper plant (Capsicum annuum L.) production, fruit quality and pollutant contents. *Spanish Journal of Agricultural Research*, *10*(1), 209–221. https://doi.org/10.5424/sjar/2012101-065-11

Gasparatos, H., & Gasparatos, A. (2020). Ecosystem services provision from urban farms in a secondary city of Myanmar, Pyin Oo Lwin. *Agriculture*, *10*(5), 1–17. https://doi.org/10.3390/agriculture10050140

Giménez, A., del Carmen Martínez-Ballesta, M., Egea-Gilabert, C., Gómez, P. A., Artés-Hernández, F., Pennisi, G., et al. (2021). Combined effect of salinity and LED lights on the yield and quality of purslane (Portulaca oleracea L.) microgreens. *Horticulturae*, *7*(7), 1–15. https://doi.org/10.3390/horticulturae7070180

González Gómez, L. G., Jiménez Arteaga, M. C., Castillo Cruz, D., Paz Martínez, I., Cambara Rodríguez, A. Y., & Falcón Rodríguez, A. (2018). Agronomic response of cucumber to application of QuitoMax under organoponic conditions. *Centro Agrícola*, *45*(3), 27–31. Retrieved from http://scielo.sld.cu/scielo.php?script=sci_arttext&pid=S0253-57852018000300027&lang=pt

Gräf, M., Stangl, R., Hood-Nowotny, R., & Kodym, A. (2020). Urban farming in indoor settings: Nitrate limits compliance check of leafy green vegetables under LED lighting. *European Journal of Horticultural Science*, *85*(5), 321–328. https://doi.org/10.17660/eJHS.2020/85.5.3

Grafius, D. R., Edmondson, J. L., Norton, B. A., Clark, R., Mears, M., Leake, J. R., et al. (2020). Estimating food production in an urban landscape. *Scientific Reports*, *10*, 1–9. https://doi.org/10.1038/s41598-020-62126-4

Grard, B. J.-P., Bel, N., Marchal, N., Madre, N., Castell, J.-F., Cambier, P., et al. (2015). Recycling urban waste as possible use for rooftop vegetable garden. *Future of Food: Journal on Food, Agriculture and Society*, *3*(1), 21–34. Retrieved from http://futureoffoodjournal.org/index.php/FOFJ/article/view/119

Grard, B. J.-P., Chenu, C., Manouchehri, N., Houot, S., Frascaria-Lacoste, N., & Aubry, C. (2018). Rooftop farming on urban waste provides many ecosystem services. *Agronomy for Sustainable Development*, *38*(2), 1–12. https://doi.org/10.1007/s13593-017-0474-2

Grard, B. J.-P., Manouchehri, N., Aubry, C., Frascaria-Lacoste, N., & Chenu, C. (2020). Potential of technosols created with urban by-products for rooftop edible production. *International Journal of Environmental Research and Public Health*, *17*(9), 1–21. https://doi.org/10.3390/ijerph17093210

Grewal, H. S., Maheshwari, B., & Parks, S. E. (2011). Water and nutrient use efficiency of a low-cost hydroponic greenhouse for a cucumber crop: An Australian case study. *Agricultural Water Management*, *98*(5), 841–846. https://doi.org/10.1016/j.agwat.2010.12.010

Gualberto, R., Rabello de Oliveira, P. S., & de Moura Guimarães, A. (2009). Adaptability and phenotypic stability of crisp lettuce cultivars in hydroponics. *Horticultura Brasileira*, *27*(1), 7–11. https://doi.org/10.1590/s0102-05362009000100002

Harada, Y., Whitlow, T. H., Bassuk, N. L., & Russell-Anelli, J. (2020). Rooftop farm soils for sustainable water and nitrogen management. *Frontiers in Sustainable Food Systems*, *4*, 1–12. https://doi.org/10.3389/fsufs.2020.00123

Harada, Y., Whitlow, T. H., Templer, P. H., Howarth, R. W., Todd Walter, M., Bassuk, N. L., & Russell-Anelli, J. (2018). Nitrogen biogeochemistry of an urban rooftop farm. *Frontiers in Ecology and Evolution*, *6*(153), 1–14. https://doi.org/10.3389/fevo.2018.00153

Hayashi, K., Abdoulaye, T., & Wakatsuki, T. (2010). Evaluation of the utilization of heated sewage sludge for peri-urban horticulture production in the Sahel of West Africa. *Agricultural Systems*, *103*(1), 36–40. https://doi.org/10.1016/j.agsy.2009.08.004

He, X., Qiao, Y., Liu, Y., Dendler, L., Yin, C., & Martin, F. (2016). Environmental impact assessment of organic and conventional tomato production in urban greenhouses of Beijing city, China. *Journal of Cleaner Production*, *134*(Part A), 251–258. https://doi.org/10.1016/j.jclepro.2015.12.004

Hemming, S., de Zwart, F., Elings, A., Petropoulou, A., & Righini, I. (2020). Cherry tomato production in intelligent greenhouses-sensors and AI for control of climate, irrigation, crop yield, and quality. *Sensors*, *20*(22), 1–30. https://doi.org/10.3390/s20226430

Hemming, S., de Zwart, F., Elings, A., Righini, I., & Petropoulou, A. (2019). Remote control of greenhouse vegetable production with artificial intelligence—greenhouse climate, irrigation, and crop production. *Sensors*, *19*(8), 1–22. https://doi.org/10.3390/s19081807

Hofkens, M., Melis, P., Laurijssen, S., Baets, D., & Van Delm, T. (2021). Four layer strawberry cultivation. *Acta Horticulturae*, *1309*(96), 663–669. https://doi.org/10.17660/ActaHortic.2021.1309.96

Holt, N., Shukla, S., Hochmuth, G., Muñoz-Carpena, R., & Ozores-Hampton, M. (2017). Transforming the food-water-energy-land-economic nexus of plasticulture production through compact bed geometries. *Advances in Water Resources*, *110*, 515–527. https://doi.org/10.1016/j.advwatres.2017.04.023

Hu, Y., Zheng, J., Kong, X., Sun, J., & Li, Y. (2019). Carbon footprint and economic efficiency of urban agriculture in Beijing——a comparative case study of conventional and home-delivery agriculture. *Journal of Cleaner Production*, *234*, 615–625. https://doi.org/10.1016/j.jclepro.2019.06.122

Hussein, M. M., Abdel-Kader, A. A., Kady, K. A., Youssef, R. A., & Alva, A. K. (2010). Sorghum response to foliar application of phosphorus and potassium with saline water irrigation. *Journal of Crop Improvement*, *24*(4), 324–336. https://doi.org/10.1080/15427528.2010.499042

Jones-Baumgardt, C., Llewellyn, D., Ying, Q., & Zheng, Y. (2019). Intensity of sole-source light-emitting diodes affects growth, yield, and quality of Brassicaceae microgreens. *HortScience*, *54*(7), 1168–1174. https://doi.org/10.21273/HORTSCI13788-18

Kadam, D., & Pathade, G. (2014). Effect of tendu (Diospyros melanoxylon RoxB.) leaf vermicompost on growth and yield of French bean (Phaseolus vulgaris L.). *International Journal of Recycling of Organic Waste in Agriculture*, *3*(1), 1–7. https://doi.org/10.1007/s40093-014-0044-4

Kalaivanan, D., Selvakumar, G., & Shankara Hebbar, S. (2020). Effects of varying N, P and K concentrations on growth, biomass, yield and nutritional quality of Zucchini squash grown under open and polyhouse soilless culture. *Indian Journal of Horticulture*, *77*(3), 496–502. https://doi.org/10.5958/0974-0112.2020.00071.7

Kamoshita, A., Ishikawa, M., Abe, J., & Imoto, H. (2007). Evaluation of water-saving rice-winter crop rotation system in a suburb of Tokyo. *Plant Production Science*, *10*(2), 219–231. https://doi.org/10.1626/pps.10.219

Kiba, D. I., Zongo, N. A., Lompo, F., Jansa, J., Compaore, E., Sedogo, P. M., & Frossard, E. (2012). The diversity of fertilization practices affects soil and crop quality in urban vegetable sites of Burkina Faso. *European Journal of Agronomy*, *38*, 12–21. https://doi.org/10.1016/j.eja.2011.11.012

Kikuchi, Y., Kanematsu, Y., Yoshikawa, N., Okubo, T., & Takagaki, M. (2018). Environmental and resource use analysis of plant factories with energy technology options: A case study in Japan. *Journal of Cleaner Production*, *186*, 703–717. https://doi.org/10.1016/j.jclepro.2018.03.110

Kim, E., Jung, J., Hapsari, G., Kang, S., Kim, K., Yoon, S., et al. (2018). Economic and environmental sustainability and public perceptions of rooftop farm versus extensive garden. *Building and Environment*, *146*, 206–215. https://doi.org/10.1016/j.buildenv.2018.09.046

Koga, N., & Tsuji, H. (2009). Effects of reduced tillage, crop residue management and manure application practices on crop yields and soil carbon sequestration on an Andisol in northern Japan. *Soil Science and Plant Nutrition*, *55*(4), 546–557. https://doi.org/10.1111/j.1747-0765.2009.00385.x

Kong, A. Y. Y., Rosenzweig, C., & Arky, J. (2015). Nitrogen dynamics associated with organic and inorganic inputs to substrate commonly used on rooftop farms. *HortScience*, *50*(6), 806–813. https://doi.org/10.21273/hortsci.50.6.806

Kurgat, B. K., Stöber, S., Mwonga, S., Lotze-Campen, H., & Rosenstock, T. S. (2018). Livelihood and climate trade-offs in Kenyan peri-urban vegetable production. *Agricultural Systems*, *160*, 79–86. https://doi.org/10.1016/j.agsy.2017.10.003

Kwon, C.-T., Heo, J., Lemmon, Z. H., Capua, Y., Hutton, S. F., Van Eck, J., et al. (2020). Rapid customization of Solanaceae fruit crops for urban agriculture. *Nature Biotechnology*, *38*, 182–188. https://doi.org/10.1038/s41587-019-0361-2

Larsen, D. H., Woltering, E. J., Nicole, C. C. S., & Marcelis, L. F. M. (2020). Response of basil growth and morphology to light intensity and spectrum in a vertical farm. *Frontiers in Plant Science*, *11*, 1–16. https://doi.org/10.3389/fpls.2020.597906

Li, S., Guo, L., Cao, C., & Li, C. (2021). Integrated assessment of carbon footprint, energy budget and net ecosystem economic efficiency from rice fields under different tillage modes in central China. *Journal of Cleaner Production*, *295*, 1–11. https://doi.org/10.1016/j.jclepro.2021.126398

Li, Y., Huang, G., Zhang, L., Gu, H., Lou, C., Zhang, H., & Liu, H. (2020). Phthalate esters (PAEs) in soil and vegetables in solar greenhouses irrigated with reclaimed water. *Environmental Science and Pollution Research*, *27*(18), 22658–22669. https://doi.org/10.1007/s11356-020-08882-2

Liang, L., Ridoutt, B. G., Lal, R., Wang, D., Wu, W., Peng, P., et al. (2019). Nitrogen footprint and nitrogen use efficiency of greenhouse tomato production in North China. *Journal of Cleaner Production*, *208*, 285–296. https://doi.org/10.1016/j.jclepro.2018.10.149

Liang, L., Ridoutt, B. G., Wu, W., Lal, R., Wang, L., Wang, Y., et al. (2019). A multi-indicator assessment of peri-urban agricultural production in Beijing, China. *Ecological Indicators*, *97*, 350–362. https://doi.org/10.1016/j.ecolind.2018.10.040

Liu, T., Yang, M., Han, Z., & Ow, D. W. (2016). Rooftop production of leafy vegetables can be profitable and less contaminated than farm-grown vegetables. *Agronomy for Sustainable Development*, *36*(41), 1–9. https://doi.org/10.1007/s13593-016-0378-6

López Labarta, P., Montejo Viamontes, J. L., Cárdenas García, Y., Piñeiro Esquivel, D., de la Caridad Vasallo Cristia, D., & Rondón Aquilar, A. (2019). Bioorganic nutritional alternatives in carrots (Daucus carota var. sativa), under intensive gardening. *Agrisost*, *25*(2), 1–5. Retrieved from https://revistas.reduc.edu.cu/index.php/agrisost/article/view/e3023

Lu, N., Maruo, T., Johkan, M., Hohjo, M., Tsukagoshi, S., Ito, Y., et al. (2012). Effects of supplemental lighting with light-emitting diodes (LEDs) on tomato yield and quality of single-truss tomato plants grown at high planting density. *Environmental Control in Biology*, *50*(1), 63–74. https://doi.org/10.2525/ecb.50.63

Ludvigson, K., Reganold, J. P., & Murphy, K. M. (2019). Sustainable intensification of quinoa production in peri-urban environments in western Washington state utilizing transplant vs. direct-seed methods. *Ciencia e Investigación Agraria*, *46*(2), 100–112. https://doi.org/10.7764/rcia.v45i2.2169

Maboko, M. M., Du Plooy, C. P., & Bertling, I. (2012). Performance of tomato cultivars in temperature and non-temperature controlled plastic tunnels. *Acta Horticulturae*, *927*(50), 405–412. https://doi.org/10.17660/actahortic.2012.927.50

Mamatha, H., Srinivasa Rao, N. K., Laxman, R. H., Shivashankara, K. S., Bhatt, R. M., & Pavithra, K. C. (2014). Impact of elevated CO2 on growth, physiology, yield, and quality of tomato (Lycopersicon esculentum Mill) cv. Arka Ashish. *Photosynthetica*, *52*(4), 519–528. https://doi.org/10.1007/s11099-014-0059-0

Manka’abusi, D., Steiner, C., Akoto-Danso, E. K., Lompo, D. J.-P., Haering, V., Werner, S., et al. (2019). Biochar application and wastewater irrigation in urban vegetable production of Ouagadougou, Burkina Faso. *Nutrient Cycling in Agroecosystems*, *115*, 263–279. https://doi.org/10.1007/s10705-019-09969-0

Martínez-Blanco, J., Muñoz, P., Antón, A., & Rieradevall, J. (2011). Assessment of tomato Mediterranean production in open-field and standard multi-tunnel greenhouse, with compost or mineral fertilizers, from an agricultural and environmental standpoint. *Journal of Cleaner Production*, *19*(9–10), 985–997. https://doi.org/10.1016/j.jclepro.2010.11.018

Matsuda, R., Kubota, C., Lucrecia Alvarez, M., & Cardineau, G. A. (2009). Biopharmaceutical protein production under controlled environments: Growth, development, and vaccine productivity of transgenic tomato plants grown hydroponically in a greenhouse. *HortScience*, *44*(6), 1594–1599. https://doi.org/10.21273/hortsci.44.6.1594

Max, J. F. J., Horst, W. J., Mutwiwa, U. N., & Tantau, H.-J. (2009). Effects of greenhouse cooling method on growth, fruit yield and quality of tomato (Solanum lycopersicum L.) in a tropical climate. *Scientia Horticulturae*, *122*(2), 179–186. https://doi.org/10.1016/j.scienta.2009.05.007

Medrano-García, P., Chipana-Rivera, R., Moreno-Pérez, M. F., & Roldán-Cañas, J. (2019). Capillary irrigation by wick in the lettuce crop ( Lactuca sativa L.) in a combined system of hydroponic solution and soil, as an alternative in urban agriculture of Bolivia. *Ingeniería Del Agua*, *23*(1), 53–63. https://doi.org/10.4995/ia.2019.10602

Michelon, N., Pennisi, G., Myint, N. O., Dall’Olio, G., Pacheco Batista, L., Cavalcante Salviano, A. A., et al. (2020). Strategies for improved yield and water use efficiency of lettuce (Lactuca sativa L.) through simplified soilless cultivation under semi-arid climate. *Agronomy*, *10*(9), 1–14. https://doi.org/10.3390/agronomy10091379

Mickens, M. A., Torralba, M., Robinson, S. A., Spencer, L. E., Romeyn, M. W., Massa, G. D., & Wheeler, R. M. (2019). Growth of red pak choi under red and blue, supplemented white, and artificial sunlight provided by LEDs. *Scientia Horticulturae*, *245*, 200–209. https://doi.org/10.1016/j.scienta.2018.10.023

Miernicki, E. A., Lovell, S. T., & Wortman, S. E. (2018). Raised beds for vegetable production in urban agriculture. *Urban Agriculture & Regional Food Systems*, *3*(1), 1–10. https://doi.org/10.2134/urbanag2018.06.0002

Miller-Robbie, L., Ramaswami, A., & Amerasinghe, P. (2017). Wastewater treatment and reuse in urban agriculture: exploring the food, energy, water, and health nexus in Hyderabad, India. *Environmental Research Letters*, *12*(7), 1–12. https://doi.org/10.1088/1748-9326/aa6bfe

Minhas, P. S., Lal, K., Yadav, R. K., Dubey, S. K., & Chaturvedi, R. K. (2015). Long term impact of waste water irrigation and nutrient rates: I. Performance, sustainability and produce quality of peri urban cropping systems. *Agricultural Water Management*, *156*, 100–109. https://doi.org/10.1016/j.agwat.2015.03.012

Mkwambisi, D. D., Fraser, E. D. G., & Dougill, A. J. (2011). Urban agriculture and poverty reduction: Evaluating how food production in cities contributes to food security, employment and income in Malawi. *Journal of International Development*, *23*(2), 181–203. https://doi.org/10.1002/jid.1657

Montero, J. I., Baeza, E., Heuvelink, E., Rieradevall, J., Muñoz, P., Ercilla, M., & Stanghellini, C. (2017). Productivity of a building-integrated roof top greenhouse in a Mediterranean climate. *Agricultural Systems*, *158*, 14–22. https://doi.org/10.1016/j.agsy.2017.08.002

Montoya-García, C. O., Volke-Haller, V. H., Santillán-Angeles, A., López-Escobar, N. F., & Trinidad-Santos, A. (2019). NH4+/NO3- ratio in the production of biomass and the nutritional content of Portulaca oleracea L. *Agrociencia*, *53*(4), 521–533.

Mowa, E., Akundabweni, L., Chimwamurombe, P., Oku, E., & Mupambwa, H. A. (2017). The influence of organic manure formulated from goat manure on growth and yield of tomato (Lycopersicum esculentum). *African Journal of Agricultural Research*, *12*(41), 3061–3067. https://doi.org/10.5897/ajar2017.12657

Muñoz, P., Paranjpe, A., Montero, J. I., & Antón, A. (2012). Cascade crops: an alternative solution for increasing sustainability of greenhouse tomato crops in Mediterranean zone. *Acta Horticulturae*, *927*(99), 801–805. https://doi.org/10.17660/actahortic.2012.927.99

Muñoz, P., Antón, A., Nuñez, M., Paranjpe, A., Ariño, J., Castells, X., et al. (2008). Comparing the environmental impacts of greenhouse versus open-field tomato production in the Mediterranean region. *Acta Horticulturae*, *801*(197), 1591–1596. https://doi.org/10.17660/ActaHortic.2008.801.197

Muñoz, P., Antón, A., Paranjpe, A., Ariño, J., & Montero, J. I. (2008). High decrease in nitrate leaching by lower N input without reducing greenhouse tomato yield. *Agronomy for Sustainable Development*, *28*(4), 489–495. https://doi.org/10.1051/agro:2008024

Muñoz, P., Flores, J. S., Antón, A., & Montero, J. I. (2017). Combination of greenhouse and open-field crop fertigation can increase sustainability of horticultural crops in the Mediterranean region. *Acta Horticulturae*, *1170*(78), 627–633. https://doi.org/10.17660/ActaHortic.2017.1170.78

Musazura, W., Odindo, A. O., Bame, I. B., & Tesfamariam, E. H. (2015). Effect of irrigation with anaerobic baffled reactor effluent on Swiss chard (Beta vulgaris cicla.) yield, nutrient uptake and leaching. *Journal of Water Reuse and Desalination*, *5*(4), 592–609. https://doi.org/10.2166/wrd.2015.011

Nabavi-Pelesaraei, A., Abdi, R., Rafiee, S., & Taromi, K. (2014). Applying data envelopment analysis approach to improve energy efficiency and reduce greenhouse gas emission of rice production. *Engineering in Agriculture, Environment and Food*, *7*(4), 155–162. https://doi.org/10.1016/j.eaef.2014.06.001

Nadal, A., Llorach-Massana, P., Cuerva, E., López-Capel, E., Montero, J. I., Josa, A., et al. (2017). Building-integrated rooftop greenhouses: An energy and environmental assessment in the Mediterranean context. *Applied Energy*, *187*, 338–351. https://doi.org/10.1016/j.apenergy.2016.11.051

Naghizadeh, M., & Hasanzadeh, R. (2012). Effect of plant density on yield, yield components, oil and protein of canola cultivars in Hajiabad. *Advances in Environmental Biology*, *6*(3), 1000–1005. Retrieved from http://www.aensiweb.com/old/aeb/2012/1000-1005.pdf

Nagle, L., Echols, S., & Tamminga, K. (2017). Food production on a living wall: Pilot study. *Journal of Green Building*, *12*(3), 23–38. https://doi.org/10.3992/1943-4618.12.3.23

Nelkin, J., & Caplow, T. (2008). Sustainable controlled environment agriculture for urban areas. *Acta Horticulturae*, *801*(48), 449–455. https://doi.org/10.17660/actahortic.2008.801.48

Nguyen, D. T. P., Lu, N., Kagawa, N., & Takagaki, M. (2019). Optimization of photosynthetic photon flux density and root-zone temperature for enhancing secondary metabolite accumulation and production of coriander in plant factory. *Agronomy*, *9*(5), 1–14. https://doi.org/10.3390/agronomy9050224

Niang, Y., Niang, S., Niassy, S., Dieng, Y., Gaye, M. L., & Diarra, K. (2012). Urban agriculture in Senegal: effect of wastewater on the agronomical performance and hygienic quality of tomato and lettuce. *International Journal of Biological and Chemical Sciences*, *6*(4), 1519–1526. https://doi.org/10.4314/ijbcs.v6i4.11

Nicholls, E., Ely, A., Birkin, L., Basu, P., & Goulson, D. (2020). The contribution of small-scale food production in urban areas to the sustainable development goals: a review and case study. *Sustainability Science*, *15*, 1585–1599. https://doi.org/10.1007/s11625-020-00792-z

Nozoe, T., Agbisit, R., Fukuta, Y., Rodriguez, R., & Yanagihara, S. (2008). Characteristics of iron tolerant rice lines developed at IRRI under field conditions. *Japan Agricultural Research Quarterly*, *42*(3), 187–192. https://doi.org/10.6090/jarq.42.187

Nwosisi, S., Nandwani, D., & Chowdhury, S. (2017). Organic vertical gardening for urban communities. *Acta Horticulturae*, *1189*(76), 399–402. https://doi.org/10.17660/ActaHortic.2017.1189.76

Nyomora, A. M. S. (2015). Effect of treated domestic wastewater as source of irrigation water and nutrients on rice performance in Morogoro, Tanzania. *Journal of Environment and Waste Management*, *2*(2), 47–55. Retrieved from https://citeseerx.ist.psu.edu/viewdoc/download?doi=10.1.1.674.1451&rep=rep1&type=pdf

Orsini, F., Fecondini, M., Mezzetti, M., Michelon, N., & Gianquinto, G. (2010). Simplified hydroponic floating systems for vegetable production in Trujillo, Peru. *Acta Horticulturae*, *881*(18), 157–161. https://doi.org/10.17660/ActaHortic.2010.881.18

Orsini, F., Gasperi, D., Marchetti, L., Piovene, C., Draghetti, S., Ramazzotti, S., et al. (2014). Exploring the production capacity of rooftop gardens (RTGs) in urban agriculture: the potential impact on food and nutrition security, biodiversity and other ecosystem services in the city of Bologna. *Food Security*, *6*, 781–792. https://doi.org/10.1007/s12571-014-0389-6

Orsini, F., Mezzetti, M., Fecondini, M., Michelon, N., & Gianquinto, G. (2010). Simplified substrate soilless culture for vegetable production in Trujillo, Peru. *Acta Horticulturae*, *881*(19), 163–167. https://doi.org/10.17660/ActaHortic.2010.881.19

Pan, T., Ding, J., Qin, G., Wang, Y., Xi, L., Yang, J., et al. (2019). Interaction of supplementary light and CO2 enrichment improves growth, photosynthesis, yield, and quality of tomato in autumn through spring greenhouse production. *HortScience*, *54*(2), 246–252. https://doi.org/10.21273/HORTSCI13709-18

Parada, F., Ercilla-Montserrat, M., Arcas-Pilz, V., Lopez-Capel, E., Carazo, N., Montero, J. I., et al. (2021). Comparison of organic substrates in urban rooftop agriculture, towards improving crop production resilience to temporary drought in Mediterranean cities. *Journal of the Science of Food and Agriculture*, *101*(14), 5888–5897. https://doi.org/10.1002/jsfa.11241

Park, J.-E., Kim, H., Kim, J., Choi, S.-J., Ham, J., Nho, C. W., & Yoo, G. (2019). A comparative study of ginseng berry production in a vertical farm and an open field. *Industrial Crops & Products*, *140*, 1–9. https://doi.org/10.1016/j.indcrop.2019.111612

Park, J.-E., Kim, J., Purevdorj, E., Son, Y.-J., Nho, C. W., & Yoo, G. (2021). Effects of long light exposure and drought stress on plant growth and glucosinolate production in pak choi (Brassica rapa subsp. chinensis). *Food Chemistry*, *340*, 1–8. https://doi.org/10.1016/j.foodchem.2020.128167

Passos, F. A., Trani, P. E., & Carvalho, C. R. L. (2015). Agronomic performance of strawberry genotypes. *Horticultura Brasileira*, *33*(2), 267–271. https://doi.org/10.1590/S0102-053620150000200021

Pennisi, G., Orsini, F., Landolfo, M., Pistillo, A., Crepaldi, A., Nicola, S., et al. (2020). Optimal photoperiod for indoor cultivation of leafy vegetables and herbs. *European Journal of Horticultural Science*, *85*(5), 329–338. https://doi.org/10.17660/eJHS.2020/85.5.4

Pennisi, G., Orsini, F., Blasioli, S., Cellini, A., Crepaldi, A., Braschi, I., et al. (2019). Resource use efficiency of indoor lettuce (Lactuca sativa L.) cultivation as affected by red:blue ratio provided by LED lighting. *Scientific Reports*, *9*, 1–11. https://doi.org/10.1038/s41598-019-50783-z

Pennisi, G., Pistillo, A., Orsini, F., Cellini, A., Spinelli, F., Nicola, S., et al. (2020). Optimal light intensity for sustainable water and energy use in indoor cultivation of lettuce and basil under red and blue LEDs. *Scientia Horticulturae*, *272*, 1–10. https://doi.org/10.1016/j.scienta.2020.109508

Pennisi, G., Pistillo, A., Orsini, F., Gianquinto, G., Fernandez, J. A., Crepaldi, A., & Nicola, S. (2020). Improved red and blue ratio in LED lighting for indoor cultivation of basil. *Acta Horticulturae*, *1271*(16), 115–118. https://doi.org/10.17660/ActaHortic.2020.1271.16

Pennisi, G., Sanyé-Mengual, E., Orsini, F., Crepaldi, A., Nicola, S., Ochoa, J., et al. (2019). Modelling environmental burdens of indoor-grown vegetables and herbs as affected by red and blue LED lighting. *Sustainability*, *11*(15), 1–21. https://doi.org/10.3390/su11154063

Pérez-Urrestarazu, L., Lobillo-Eguíbar, J., Fernández-Cañero, R., & Fernández-Cabanás, V. M. (2019). Suitability and optimization of FAO’s small-scale aquaponics systems for joint production of lettuce (Lactuca sativa) and fish (Carassius auratus). *Aquacultural Engineering*, *85*, 129–137. https://doi.org/10.1016/j.aquaeng.2019.04.001

Perrin, A., Basset-Mens, C., Huat, J., & Gabrielle, B. (2017). The variability of field emissions is critical to assessing the environmental impacts of vegetables: A Benin case-study. *Journal of Cleaner Production*, *153*, 104–113. https://doi.org/10.1016/j.jclepro.2017.03.159

Perrin, A., Basset-Mens, C., Huat, J., & Yehouessi, W. (2015). High environmental risk and low yield of urban tomato gardens in Benin. *Agronomy for Sustainable Development*, *35*(1), 305–315. https://doi.org/10.1007/s13593-014-0241-6

Petran, A., Hoover, E., Hayes, L., & Poppe, S. (2017). Yield and quality characteristics of day-neutral strawberry in the United States Upper Midwest using organic practices. *Biological Agriculture and Horticulture*, *33*(2), 73–88. https://doi.org/10.1080/01448765.2016.1188152

Pourias, J., Duchemin, E., & Aubry, C. (2015). Products from urban collective gardens: Food for thought or for consumption? Insights from Paris and Montreal. *Journal of Agriculture, Food Systems, and Community Development*, *5*(2), 175–199. https://doi.org/10.5304/jafscd.2015.052.005

Pramanick, K. K., Kishore, D. K., Watpade, S., & Sharma, Y. P. (2017). Prospects of strawberry cultivation in urban areas of India. *Acta Horticulturae*, *1181*(13), 99–106. https://doi.org/10.17660/ActaHortic.2017.1181.13

Raimondi, G., Orsini, F., Maggio, A., De Pascale, S., & Barbieri, G. (2006). Yield and quality of hydroponically grown sweet basil cultivars. *Acta Horticulturae*, *723*(48), 357–363. https://doi.org/10.17660/actahortic.2006.723.48

Rakocy, J. E., Shultz, R. C., Bailey, D. S., & Thoman, E. S. (2004). Aquaponic production of tilapia and basil: Comparing a batch and staggered cropping system. *Acta Horticulturae*, *648*(8), 63–69. https://doi.org/10.17660/ActaHortic.2004.648.8

Rasco, E. T., Mangubat, J. R., Burgonio, A. B., Logrono, M. L., Villegas, V. N., & Fernandez, E. C. (2010). Agronomic performance and Asiatic corn borer resistance of tropical converted transgenic corn hybrids containing the truncated Cry1A(b) gene (Bt-11) in Davao City, Philippines. *Philippine Journal of Crop Science*, *35*(1), 1–15. Retrieved from https://www.cabi.org/gara/abstract/20103139946

Reeves, J., Cheng, Z., Kovach, J., Kleinhenz, M. D., & Grewal, P. S. (2014). Quantifying soil health and tomato crop productivity in urban community and market gardens. *Urban Ecosystems*, *17*, 221–238. https://doi.org/10.1007/s11252-013-0308-1

Ribas-Agustí, A., Seda, M., Sarraga, C., Montero, J. I., Castellari, M., & Muñoz, P. (2017). Municipal solid waste composting: Application as a tomato fertilizer and its effect on crop yield, fruit quality and phenolic content. *Renewable Agriculture and Food Systems*, *32*(4), 358–365. https://doi.org/10.1017/S1742170516000296

Richards, P. J., Farrell, C., Tom, M., Williams, N. S. G., & Fletcher, T. D. (2015). Vegetable raingardens can produce food and reduce stormwater runoff. *Urban Forestry & Urban Greening*, *14*(3), 646–654. https://doi.org/10.1016/j.ufug.2015.06.007

Rodríguez-Delfín, A., Hoyos, M., Chang, M., Castro, G., Barreda, E., & Tamo, J. (2005). Evaluation of growth and yield of “roja arequipeña” onion grown in two natural substrates. *Acta Horticulturae*, *697*(65), 505–510. https://doi.org/10.17660/actahortic.2005.697.65

Rosin, K. G., Kumar, S., Patel, N., Lal, K., Kaur, R., & Sharma, V. K. (2020). Effect of improved land and water management strategies on crop productivity and soil fertility in wastewater irrigated eggplant (Solanum melongena). *Indian Journal of Agricultural Sciences*, *90*(10), 1959–1963. Retrieved from http://epubs.icar.org.in/ejournal/index.php/IJAgS/article/view/107973

Rothwell, A., Ridoutt, B., Page, G., & Bellotti, W. (2015). Feeding and housing the urban population: Environmental impacts at the peri-urban interface under different land-use scenarios. *Land Use Policy*, *48*, 377–388. https://doi.org/10.1016/j.landusepol.2015.06.017

Rufí-Salís, M., Petit-Boix, A., Villalba, G., Gabarrell, X., & Leipold, S. (2021). Combining LCA and circularity assessments in complex production systems: the case of urban agriculture. *Resources, Conservation & Recycling*, *166*, 1–12. https://doi.org/10.1016/j.resconrec.2020.105359

Rufí-Salís, M., Petit-Boix, A., Villalba, G., Sanjuan-Delmás, D., Parada, F., Ercilla-Montserrat, M., et al. (2020). Recirculating water and nutrients in urban agriculture: An opportunity towards environmental sustainability and water use efficiency? *Journal of Cleaner Production*, *261*, 1–11. https://doi.org/10.1016/j.jclepro.2020.121213

Saeidi, M., Moradi, F., & Abdoli, M. (2017). Impact of drought stress on yield, photosynthesis rate, and sugar alcohols contents in wheat after anthesis in semiarid region of Iran. *Arid Land Research and Management*, *31*(2), 204–218. https://doi.org/10.1080/15324982.2016.1260073

Saengtharatip, S., Joshi, J., Zhang, G., Takagaki, M., Kozai, T., & Yamori, W. (2021). Optimal light wavelength for a novel cultivation system with a supplemental upward lighting in plant factory with artificial lighting. *Environmental Control in Biology*, *59*(1), 21–27. https://doi.org/10.2525/ECB.59.21

Safi, Z., Dossa, L. H., & Buerkert, A. (2011). Economic analysis of cereal, vegetable and grape production systems in urban and peri-urban agriculture of Kabul, Afghanistan. *Experimental Agriculture*, *47*(4), 705–716. https://doi.org/10.1017/S0014479711000482

Sallume, M. O., Abood, M. A., Hamdi, G. J., & Sarheed, B. R. (2020). Influence of foliar fertilization of amino decanate® on growth and yield of eggplant (Solanum melongena) under water stress condition. *Research on Crops*, *21*(3), 557–562. https://doi.org/10.31830/2348-7542.2020.087

Sangare, S. K., Compaore, E., Buerkert, A., Vanclooster, M., Sedogo, M. P., & Bielders, C. L. (2012). Field-scale analysis of water and nutrient use efficiency for vegetable production in a West African urban agricultural system. *Nutrient Cycling in Agroecosystems*, *92*, 207–224. https://doi.org/10.1007/s10705-012-9484-2

Sanjuan-Delmás, D., Llorach-Massana, P., Nadal, A., Ercilla-Montserrat, M., Muñoz, P., Montero, J. I., et al. (2018). Environmental assessment of an integrated rooftop greenhouse for food production in cities. *Journal of Cleaner Production*, *177*, 326–337. https://doi.org/10.1016/j.jclepro.2017.12.147

Santosa, M., & Yulianto, N. (2020). Growth and yield of beans (Phaseolus radiatus L) planted in Malang urban farming. *Bioscience Research*, *17*(2), 1041–1050.

Santoso, M., & Anggita, L. (2019). The performance of Lebat-3 beans (Phaseolus vulgaris L.) grown in organic urban farming system. *Bioscience Research*, *16*(2), 1243–1250.

Sanyé-Mengual, E., Gasperi, D., Michelon, N., Orsini, F., Ponchia, G., & Gianquinto, G. (2018). Eco-efficiency assessment and food security potential of home gardening: A case study in Padua, Italy. *Sustainability*, *10*(7), 1–25. https://doi.org/10.3390/su10072124

Särkkä, L. E., Jokinen, K., Ottosen, C. O., & Kaukoranta, T. (2017). Effects of HPS and LED lighting on cucumber leaf photosynthesis, light quality penetration and temperature in the canopy, plant morphology and yield. *Agricultural and Food Science*, *26*(2), 102–110. https://doi.org/10.23986/afsci.60293

Segura, M. L., Contreras París, J. I., Plaza, B. M., & Lao, M. T. (2012). Assessment of the nitrogen and potassium fertilizer in green bean irrigated with disinfected urban wastewater. *Communications in Soil Science and Plant Analysis*, *43*(1–2), 426–433. https://doi.org/10.1080/00103624.2011.638604

Segura, M. L., Contreras, J., García-Delgado, C., & Eymar, E. (2010). Use of disinfected wastewater for pepper fertigation in Almeria (Spain): Evaluation of chemical risks in soil and leachates. *Acta Horticulturae*, *852*(34), 275–282. https://doi.org/10.17660/actahortic.2010.852.34

Semilla, M. G., Agulto, I., Espino Jr, A., & Sicat, E. (2018). Indoor production of loose-leaf lettuce (Lactuca sativa L.) using artificial lights and cooling system in tropical lowland. *MATEC Web of Conferences*, *192*, 1–4. https://doi.org/10.1051/matecconf/201819203016

Shrestha, P., Small, G. E., & Kay, A. (2020). Quantifying nutrient recovery efficiency and loss from compost-based urban agriculture. *PLoS ONE*, *15*(4), 1–15. https://doi.org/10.1371/journal.pone.0230996

Shumba, A., Dunjana, N., Nyamasoka, B., Nyamugafata, P., Madyiwa, S., & Nyamangara, J. (2020). Maize (Zea mays) yield and its relationship to soil properties under integrated fertility, mulch and tillage management in urban agriculture. *South African Journal of Plant and Soil*, *37*(2), 120–129. https://doi.org/10.1080/02571862.2019.1678686

Simon, S., Assogba Komlan, F., Adjaïto, L., Mensah, A., Coffi, H. K., Ngouajio, M., & Martin, T. (2014). Efficacy of insect nets for cabbage production and pest management depending on the net removal frequency and microclimate. *International Journal of Pest Management*, *60*(3), 208–216. https://doi.org/10.1080/09670874.2014.956844

Small, G., Sisombath, B., Reuss, L., Henry, R., & Kay, A. (2017). Assessing how the ratio of barley mash to wood chips in compost affects rates of microbial processing and subsequent vegetable yield. *Compost Science & Utilization*, *25*(4), 272–281. https://doi.org/10.1080/1065657X.2017.1329038

Sobczak, A., Kowalczyk, K., Gajc-Wolska, J., Kowalczyk, W., & Niedzinska, M. (2020). Growth, yield and quality of sweet pepper fruits fertilized with polyphosphates in hydroponic cultivation with LED lighting. *Agronomy*, *10*(10), 1–15. https://doi.org/10.3390/agronomy10101560

Song, J., Chen, Y., Mao, Q., Hong, X., & Yan, C. (2021). The effect of early season rice varieties and establishment methods on greenhouse gas emissions in southeast China. *Cereal Research Communications*, *49*, 567–576. https://doi.org/10.1007/s42976-021-00134-4

Spalholz, H., Perkins-Veazie, P., & Hernández, R. (2020). Impact of sun-simulated white light and varied blue:red spectrums on the growth, morphology, development, and phytochemical content of green- and red-leaf lettuce at different growth stages. *Scientia Horticulturae*, *264*, 1–12. https://doi.org/10.1016/j.scienta.2020.109195

Splawski, C. E., Regnier, E. E., Kent Harrison, S., Bennett, M. A., & Metzger, J. D. (2016). Weed suppression in pumpkin by mulches composed of organic municipal waste materials. *HortScience*, *51*(6), 720–726. https://doi.org/10.21273/hortsci.51.6.720

Steiner, C., Bellwood-Howard, I., Häring, V., Tonkudor, K., Addai, F., Atiah, K., et al. (2018). Participatory trials of on-farm biochar production and use in Tamale, Ghana. *Agronomy for Sustainable Development*, *38*, 1–10. https://doi.org/10.1007/s13593-017-0486-y

Su, Y.-L., Wang, Y.-F., & Ow, D. W. (2020). Increasing effectiveness of urban rooftop farming through reflector-assisted double-layer hydroponic production. *Urban Forestry & Urban Greening*, *54*, 1–9. https://doi.org/10.1016/j.ufug.2020.126766

Sullivan, C., Hallaran, T., Sogorka, G., & Weinkle, K. (2015). An evaluation of conventional and subirrigated planters for urban agriculture: Supporting evidence. *Renewable Agriculture and Food Systems*, *30*(1), 55–63. https://doi.org/10.1017/S1742170514000131

Sun, H., Zhang, H., Xiao, H., Shi, W., Müller, K., Van Zwieten, L., & Wang, H. (2019). Wheat straw biochar application increases ammonia volatilization from an urban compacted soil giving a short-term reduction in fertilizer nitrogen use efficiency. *Journal of Soils and Sediments*, *19*(4), 1624–1631. https://doi.org/10.1007/s11368-018-2169-y

Swann, K., Hadley, P., Else, M. A., Pearson, S., Badiee, A., & Twitchen, C. (2021). The effect of light intensity and duration on yield and quality of everbearer and June-bearer strawberry cultivars in a LED lit multi-tiered vertical growing system. *Acta Horticulturae*, *1309*(52), 359–366. https://doi.org/10.17660/ActaHortic.2021.1309.52

Takahashi, M., Ohishi, M., Sato, F., Okada, K., & Sasaki, H. (2021). Enlarging broccoli (Brassica oleracea L. var. italica) heads by extending the growing period and sparse planting to increase floret yield. *The Horticulture Journal*, *90*(1), 75–84. https://doi.org/10.2503/hortj.UTD-241

Tang, Q., Ti, C., Xia, L., Xia, Y., Wei, Z., & Yan, X. (2019). Ecosystem services of partial organic substitution for chemical fertilizer in a peri-urban zone in China. *Journal of Cleaner Production*, *224*, 779–788. https://doi.org/10.1016/j.jclepro.2019.03.201

Tavan, M., Wee, B., Brodie, G., Fuentes, S., Pang, A., & Gupta, D. (2021). Optimizing sensor-based irrigation management in a soilless vertical farm for growing microgreens. *Frontiers in Sustainable Food Systems*, *4*, 1–12. https://doi.org/10.3389/fsufs.2020.622720

Taylor, J. R. (2020). Modeling the potential productivity of urban agriculture and its impacts on soil quality through experimental research on scale-appropriate systems. *Frontiers in Sustainable Food Systems*, *4*(89), 1–18. https://doi.org/10.3389/fsufs.2020.00089

Tewolde, F. T., Lu, N., Shiina, K., Maruo, T., Takagaki, M., Kozai, T., & Yamori, W. (2016). Nighttime supplemental LED inter-lighting improves growth and yield of single-truss tomatoes by enhancing photosynthesis in both winter and summer. *Frontiers in Plant Science*, *7*, 1–10. https://doi.org/10.3389/fpls.2016.00448

Touliatos, D., Dodd, I. C., & McAinsh, M. (2016). Vertical farming increases lettuce yield per unit area compared to conventional horizontal hydroponics. *Food and Energy Security*, *5*(3), 184–191. https://doi.org/10.1002/fes3.83

Tuzel, Y., Gul, A., Tuncay, O., Anac, D., Madanlar, N., Yoldas, Z., et al. (2005). Organic cucumber production in the greenhouse: A case study from Turkey. *Renewable Agriculture and Food Systems*, *20*(4), 206–213. https://doi.org/10.1079/raf2005105

Ulm, F., Avelar, D., Hobson, P., Penha-Lopes, G., Dias, T., Máguas, C., & Cruz, C. (2019). Sustainable urban agriculture using compost and an open-pollinated maize variety. *Journal of Cleaner Production*, *212*, 622–629. https://doi.org/10.1016/j.jclepro.2018.12.069

Van Ginkel, S. W., Igou, T., & Chen, Y. (2017). Energy, water and nutrient impacts of California-grown vegetables compared to controlled environmental agriculture systems in Atlanta, GA. *Resources, Conservation and Recycling*, *122*, 319–325. https://doi.org/10.1016/j.resconrec.2017.03.003

Varela, A., Sandoval-Albán, A., Muñoz, M., Gómez Gómez, A., Bogoya, J. M., & Combariza, G. (2021). Evaluation of green roof structures and substrates for Lactuca sativa L. in tropical conditions. *Urban Forestry & Urban Greening*, *60*, 1–9. https://doi.org/10.1016/j.ufug.2021.127063

Vogl, C. R., Axmann, P., & Vogl-Lukasser, B. (2004). Urban organic farming in Austria with the concept of Selbsternte ('self-harvest’): An agronomic and socio-economic analysis. *Renewable Agriculture and Food Systems*, *19*(2), 67–79. https://doi.org/10.1079/rafs200062

Wahid, A., Milne, E., Shamsi, S. R. A., Ashmore, M. R., & Marshall, F. M. (2001). Effects of oxidants on soybean growth and yield in the Pakistan Punjab. *Environmental Pollution*, *113*(3), 271–280. https://doi.org/10.1016/S0269-7491(00)00190-1

Wan, N.-F., Cai, Y.-M., Shen, Y.-J., Ji, X.-Y., Wu, X.-W., Zheng, X.-R., et al. (2018). Increasing plant diversity with border crops reduces insecticide use and increases crop yield in urban agriculture. *ELife*, *7*, 1–21. https://doi.org/10.7554/eLife.35103

Whittinghill, L. J., Rowe, D. B., & Cregg, B. M. (2013). Evaluation of vegetable production on extensive green roofs. *Agroecology and Sustainable Food Systems*, *37*(4), 465–484. https://doi.org/10.1080/21683565.2012.756847

Widaryanto, E., Azizah, N., & Fitriyah, N. L. (2017). Effect of water deficit and planting materials on growth and yield of watercress (Nasturtium officinale) in urban farming system. *Bioscience Research*, *14*(2), 170–177.

Wiggins, Z., Akaeze, O., Nandwani, D., & Witcher, A. (2020). Substrate properties and fertilizer rates on yield responses of lettuce in a vertical growth system. *Sustainability*, *12*(16), 1–8. https://doi.org/10.3390/su12166465

Wijitkosum, S., & Jiwnok, P. (2019). Effect of biochar on Chinese kale and carbon storage in an agricultural area on a high rise building. *AIMS Agriculture and Food*, *4*(1), 177–193. https://doi.org/10.3934/AGRFOOD.2019.1.177

Win, E. P., Win, K. K., Bellingrath-Kimura, S. D., & Oo, A. Z. (2020). Greenhouse gas emissions, grain yield and water productivity: a paddy rice field case study based in Myanmar. *Greenhouse Gases: Science and Technology*, *10*(5), 884–897. https://doi.org/10.1002/ghg.2011

Wittmann, S., Jüttner, I., & Mempel, H. (2020). Indoor farming marjoram production—quality, resource efficiency, and potential of application. *Agronomy*, *10*(11), 1–17. https://doi.org/10.3390/agronomy10111769

Wortman, S. E. (2015). Crop physiological response to nutrient solution electrical conductivity and pH in an ebb-and-flow hydroponic system. *Scientia Horticulturae*, *194*, 34–42. https://doi.org/10.1016/j.scienta.2015.07.045

Xie, J., Yu, J., Chen, B., Feng, Z., Li, J., Zhao, C., et al. (2017). *Facility Cultivation Systems “设施农业”: A Chinese Model for the Planet*. *Advances in Agronomy* (1st ed., Vol. 145). Elsevier Inc. https://doi.org/10.1016/bs.agron.2017.05.005

Zabel, P., Bornemann, G., Tajmar, M., & Schubert, D. (2019). Yield of dwarf tomatoes grown with a nutrient solution based on recycled synthetic urine. *Life Sciences in Space Research*, *20*, 62–71. https://doi.org/10.1016/j.lssr.2019.01.001

Zaragoza, G., Buchholz, M., Jochum, P., & Pérez-Parra, J. (2007). Watergy project: Towards a rational use of water in greenhouse agriculture and sustainable architecture. *Desalination*, *211*(1–3), 296–303. https://doi.org/10.1016/j.desal.2006.03.599

Zaragoza, G., Buendía, D., Meca, D., Pérez-Parra, J., & Buchholz, M. (2008). Experiences in cultivation inside the watergy prototype of a closed greenhouse for semi-arid regions. *Acta Horticulturae*, *801*(90), 773–780. https://doi.org/10.17660/ActaHortic.2008.801.90

Zou, J., Zhou, C., Xu, H., Cheng, R., Yang, Q., & Li, T. (2020). The effect of artificial solar spectrum on growth of cucumber and lettuce under controlled environment. *Journal of Integrative Agriculture*, *19*(8), 2027–2034. https://doi.org/10.1016/S2095-3119(20)63209-9
