# Supplementary material for: How Much Food Can We Grow in Urban Areas? Food Production and Crop Yields of Urban Agriculture: A Meta‐Analysis
Source: Earths Future. 2022 Aug 26;10(8):e2022EF002748. doi: 10.1029/2022EF002748 (PMC9540868; doi:10.1029/2022EF002748)
Supplement: Supplementary file 2 — Table S1 [file EFT2-10-e2022EF002748-s002.docx]

**Table S1**. Mean crop yields per growing cycle of urban agriculture by disaggregated crop category (data from this meta-analysis).

| Aggregated crop categories | Disaggregated crop categories | Mean crop yields (kg m^-2^ cycle^-1^) | Number of observations |
| --- | --- | --- | --- |
| Cereals | Barley | 0.42 | 5 |
|  | Cereals nes | 1.8 | 16 |
|  | Maize | 0.55 | 93 |
|  | Millet | 2.0 | 2 |
|  | Paddy rice | 0.61 | 124 |
|  | Quinoa | 0.23 | 8 |
|  | Sorghum | 0.68 | 11 |
|  | Wheat | 0.44 | 62 |
| Fibre crops primary | Bastfibres, other | 0.59 | 4 |
|  | Jute | 0.34 | 9 |
| Fruit primary | Apples | 0.96 | 2 |
|  | Avocados | 0.33 | 1 |
|  | Bananas | 0.36 | 1 |
|  | Berries nes | 0.56 | 4 |
|  | Blueberries | 1.3 | 1 |
|  | Cherries | 8.4 | 1 |
|  | Currants | 0.76 | 2 |
|  | Fruit, tropical fresh nes | 0.17 | 1 |
|  | Gooseberries | 1.9 | 1 |
|  | Mangoes, mangosteens, guavas | 0.32 | 1 |
|  | Melons, other (including cantaloupes) | 2.3 | 2 |
|  | Papayas | 0.20 | 1 |
|  | Peaches and nectarines | 4.0 | 1 |
|  | Pears | 0.71 | 2 |
|  | Persimmons | 0.76 | 1 |
|  | Plums and sloes | 0.50 | 3 |
|  | Raspberries | 1.1 | 1 |
|  | Strawberries | 2.5 | 70 |
|  | Watermelons | 5.2 | 2 |
| Oilcrops | Rapeseed | 1.3 | 16 |
|  | Soybeans | 0.26 | 12 |
| Pulses | Chickpeas | 0.050 | 1 |
| Roots and tubers | Potatoes | 4.0 | 14 |
|  | Roots and tubers nes | 6.0 | 4 |
|  | Sweet potatoes | 1.8 | 6 |
| Sugar crops primary | Sugar beet | 5.3 | 8 |
| Vegetables primary | Anise, badian, fennel, coriander | 1.7 | 24 |
|  | Artichokes | 2.1 | 1 |
|  | Asparagus | 0.20 | 1 |
|  | Aubergines | 3.3 | 37 |
|  | Beans | 1.5 | 85 |
|  | Cabbages and other brassicas | 3.6 | 238 |
|  | Carrots and turnips | 4.7 | 41 |
|  | Cauliflowers and broccoli | 1.9 | 134 |
|  | Chillies and peppers | 5.5 | 69 |
|  | Cucumbers and gherkins | 17 | 58 |
|  | Garlic | 2.3 | 13 |
|  | Leeks and other alliaceous vegetables | 2.7 | 3 |
|  | Lettuce and chicory | 3.8 | 344 |
|  | Okra | 1.1 | 15 |
|  | Onions and shallots | 1.8 | 20 |
|  | Peas | 1.8 | 8 |
|  | Peppermint | 3.7 | 1 |
|  | Pumpkins, squash and gourds | 4.0 | 37 |
|  | Spices nes | 1.5 | 1 |
|  | Spinach | 3.6 | 29 |
|  | Tomatoes | 8.7 | 208 |
|  | Vegetables, fresh nes | 4.2 | 202 |
